# The Indoor Microbiome: Sampling, Analysis and Emerging Trends

**DOI:** 10.1111/1758-2229.70272

**Published:** 2026-04-07

**Authors:** Iva Šunić, Jelena Šarac, Dubravka Havaš Auguštin, Sofya Pozdniakova, Robert M. W. Ferguson, Matijana Jergović, David Visentin, Sílvia Borràs, Elizabeth Archer, Drew K. Henderson, Sandra Vitko, Adna Ašić, Anja Bošnjaković, Željka Maglica, Carla Viegas, Natalija Novokmet, Nina Karlović, Damir Marjanović, Adam Muszyński, Yuxi Liu, Piia Karisola, Harri Alenius, Lukasz Krych, Mario Lovrić

**Affiliations:** ^1^ Institute for Anthropological Research Zagreb Croatia; ^2^ Faculty of Biotechnology and Drug Development University of Rijeka Rijeka Croatia; ^3^ AIRLAB, Barcelona Institute for Global Health Barcelona Spain; ^4^ School of Life Sciences University of Essex Colchester UK; ^5^ Andrija Štampar Teaching Institute of Public Health Zagreb Croatia; ^6^ Department of Biology, Faculty of Science University of Zagreb Zagreb Croatia; ^7^ Research Institute Verlab for Biomedical Engineering Medical Devices and Artificial Intelligence Sarajevo Bosnia and Herzegovina; ^8^ H&TRC Health and Technology Research Center, ESTeSL Escola Superior de Tecnologia da Saúde Instituto Politécnico de Lisboa Lisbon Portugal; ^9^ NOVA National School of Public Health, Public Health Research Centre, Comprehensive Health Research Center, CHRC, REAL, CCAL NOVA University Lisbon Lisbon Portugal; ^10^ Faculty of Environmental Engineering Warsaw University of Technology Warsaw Poland; ^11^ Human Microbiome Research (HUMI), Faculty of Medicine University of Helsinki Helsinki Finland; ^12^ Institute of Environmental Medicine Karolinska Institutet Stockholm Sweden; ^13^ Department of Food Science, Faculty of Science University of Copenhagen Frederiksberg Denmark; ^14^ The Lisbon Council Brussels Belgium

**Keywords:** dust microbiome, indoor air quality, methodological harmonisation, sampling methods, sequencing methods

## Abstract

Indoor spaces contain diverse microbial communities that shape human health. These microorganisms are particularly relevant to respiratory diseases, including asthma and allergies. Despite growing recognition of the importance of indoor microbial exposures, research in this field is slowed by differences in methods. These inconsistencies make it difficult to compare results and draw conclusions. This systematic review analyses 106 studies published between 2000 and 2025 that investigated indoor microbiomes in dust, air, and other matrices across homes, schools, and other built environments. We assessed sampling strategies, DNA extraction protocols, sequencing technologies, and bioinformatic pipelines, identifying trends, inconsistencies, and areas requiring harmonisation. Passive sampling, particularly dust collection, was the most common approach, while Illumina‐based 16S rRNA and ITS amplicon sequencing dominated molecular analyses. However, variations in targeted gene regions, extraction kits, and analytical tools limited cross‐study comparability. Ecological findings revealed consistent detection of bacterial taxa such as *Staphylococcus*, *Streptococcus*, and *Corynebacterium*, and fungal taxa including *Cladosporium*, *Aspergillus*, and *Penicillium*, with diversity shaped by building characteristics, ventilation, humidity, occupancy, and presence of pets. This review highlights the need for standardised protocols in indoor microbiome research to facilitate reproducibility, enable meta‐analyses, and inform health‐related guidelines for indoor environments.

## Graphical abstract

This systematic review of 106 studies highlights indoor microbiome sampling and analysis methods, dominant bacterial and fungal taxa, and key environmental drivers. Despite progress, methodological inconsistencies hinder comparability, emphasising the need for standardised approaches to advance indoor microbiome research and its influence on health.

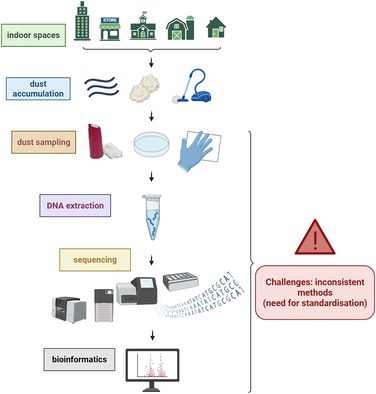

## 1 Introduction

Daily, we are exposed to vast amounts of air pollutants, including particulate matter (PM), dust particles, chemicals, gases, as well as biological particles (“bioaerosols”) such as allergens, pollen, and microorganisms. Air pollution represents an additional important risk for increased mortality and morbidity, particularly concerning cardio/cerebrovascular and respiratory diseases, as well as metabolic and neurological disorders (Cai et al. 2023; Cobbold et al. 2022; de Bont et al. 2022; Delgado‐Saborit et al. 2021; Dijkhoff et al. 2020; Dowlatabadi et al. 2024; European Environment Agency 2024; Eze et al. 2015; Font‐Ribera et al. 2023; GBD 2021 HAP Collaborators 2025; Gilbert and Hartmann 2024; Juginović et al. 2021; Keleb et al. 2025; Kim et al. 2025; Lane et al. 2025; Oh et al. 2025; Soares et al. 2024; Zhao et al. 2024; European Environment Agency 2025). While outdoor air pollution has been extensively studied, indoor air quality (IAQ) has garnered increasing attention due to the significant amount of time individuals spend indoors, in homes, schools, kindergartens, workplaces, residencies, hospitals, and other enclosed environments (WHO 2024; Lovrić et al. 2025; Pillarisetti et al. 2022; Vilcins et al. 2024). Common indoor pollutants encompass both chemical and biological agents (Furst et al. 2025; Martins et al. 2025). Chemical pollutants include volatile organic compounds (VOCs, such as formaldehyde) (Račić et al. 2025), as well as tobacco smoke. Biological pollutants comprise dust mites, mould, pollen, pet dander, and microbial agents (Tran et al. 2020). Environmental parameters can significantly shape IAQ; for example, high humidity/damp indoor conditions particularly favour the proliferation of moulds and bacteria (WHO 2009), turning the indoor environment into a reservoir of allergens, mycotoxins, endotoxins, and other airborne irritants that may exacerbate asthma and other respiratory conditions (WHO 2009; Jie et al. 2011; Louisias et al. 2019; Vandenborght et al. 2021). However, relative humidity (RH) exerts contrasting effects on the survival of bacteria and viruses in the environment, particularly in droplets. Generally, bacterial viability decreases with lower RH, while viruses tend to remain viable at both low (< 33%) and very high (100%) RH levels, but their survival decreases at intermediate RHs. This difference is attributed to distinct inactivation mechanisms, highlighting a fundamental divergence in how humidity modulates microbial persistence (Lin and Marr 2020; Oswin et al. 2022).

The indoor environment, hence, serves as a reservoir for diverse microbial communities, collectively referred to as the indoor microbiome. Most indoor microorganisms derive from transient sources, such as outdoor air, building materials, human occupants, pets, and ventilation systems (Gilbert and Stephens 2018; Hoisington et al. 2023). Consequently, the composition of the indoor microbiome largely reflects environmental inputs rather than the presence of stable endemic taxa. These indoor/outdoor interactions, together with the microbial dynamics occurring indoors, influence human exposure and health, particularly given that modern populations spend approximately 80%–90% of their time in the built environment (Gilbert and Stephens 2018; Hoisington et al. 2023; Yang et al. 2023). Recent perspectives suggest that by understanding and intentionally managing these microbial interactions, it may be possible to design health‐promoting indoor environments—so‐called “probiotic homes”—that support beneficial microbial communities and reduce risks associated with dysbiotic indoor microbiomes (Bourzac 2025). The indoor microbiome can serve as a potential source of microbial colonisers for a developing respiratory tract in children, directly impacting their immune system maturation (Gupta et al. 2020; Man et al. 2017; Mortensen et al. 2016). Respiratory diseases are common in early childhood and are often associated with an immature immune system. Recent studies have shown that early‐life exposure to a diverse microbiome, including that found in indoor air and dust, is crucial for shaping immune homeostasis (Gensollen et al. 2016; Rook 2023). A well‐balanced microbial exposure during critical developmental windows supports immune tolerance and may protect against the development of childhood asthma and atopic diseases, whereas disruption in this interaction may contribute to immune dysregulation (Rook 2023; Bisgaard et al. 2014; Topalušić et al. 2022). These findings highlight the importance of indoor microbial diversity in early life and underscore the need for further research on how IAQ contributes to long‐term immune and respiratory health. The importance of the microbial environment's impact on health has been increasingly recognised, prompting more research on public spaces such as daycares, schools, offices, homes, university classrooms, sports facilities, museums, subway stations, various facilities, and public restrooms, as well as in occupational environments such as farming, composting, home healthcare, and industrial settings (Vandenborght et al. 2021; Cox et al. 2022; Dalton et al. 2024; Ege et al. 2011; Guo et al. 2020; Kettleson et al. 2015; Kirjavainen et al. 2019; Shan et al. 2019; Tischer et al. 2016; Viegas et al. 2017). Research on respiratory health has revealed a link between asthma and other respiratory conditions, where the presence or absence of specific taxa influences respiratory health (Cox et al. 2022; Tischer et al. 2016). Exposure to a greater diversity of microbiomes in early childhood can help prevent sensitivity to aeroallergens (Kirjavainen et al. 2019; Tischer et al. 2016). On the other hand, longitudinal studies have confirmed that phylogenetic differences in the dust microbiome in infants' homes at 2 months old are associated with a higher risk of asthma around the age of 10. These results suggest that bacterial communities are more closely linked to asthma protection than individual bacterial species or microbial quantity (Karvonen et al. 2019). Overall, the findings underscore the crucial role of microbial diversity and composition in early life environments in shaping long‐term respiratory health.

The growing awareness of the impact of indoor air on human health has catalysed research efforts aimed at better understanding and promoting healthier built environments. The IDEAL Cluster (Lovrić et al. 2025), a Horizon Europe‐funded consortium, brings together multidisciplinary projects across Europe to advance knowledge on indoor microbial exposures. Key aims include methodological harmonisation, interdisciplinary collaboration, and the development of standardised, data‐driven approaches. A key issue highlighted by the consortium is the limited evidence base for establishing guidelines for exposure to biological air pollutants (Cervantes et al. 2025), which the IDEAL Cluster aims to address empirically. Additionally, the heterogeneity of sampling and analytical methods used to collect data makes the intercomparison of results challenging (Whitby et al. 2022).

Despite increased research activity, progress in understanding the role of the indoor microbiome in health remains limited by the heterogeneity of study designs and analytical approaches. Recent literature has also underscored substantial methodological inconsistencies in microbiome sampling, including discrepancies in sampling strategies, transport conditions, storage temperatures, elution protocols, and analytical assays (Dias et al. 2024). These gaps make it difficult to synthesise findings, assess health risks, and develop meaningful regulatory standards, subsequently hindering cross‐study comparisons and evidence synthesis, and ultimately impeding the development of actionable health guidelines. Addressing this gap requires a systematic assessment of current methodological practices in indoor microbiome research. Therefore, the objective of this paper is to systematically review and evaluate the methodological approaches employed in indoor microbiome research, with a focus on sampling strategies and methods, DNA extraction and sequencing protocols, and bioinformatic analysis tools, to identify trends, inconsistencies, and areas requiring consensus/harmonisation to allow the comparability and reliability of future studies. This review does not primarily seek to assess health outcomes, but rather to:

1. Map and classify the sampling methods employed (active, such as air sampling via filtration or impaction, and passive, such as surface swabs) and materials recovered.
2. Assess how the sampling methods employed influence the data comparability.
3. Examine used DNA extraction protocols and sequencing techniques (e.g., 16S rRNA and ITS genes amplicon sequencing, shotgun metagenomics), along with commonly used gene regions and platforms (e.g., Illumina MiSeq).
4. Identify analytical tools and pipelines used for microbial community analysis (e.g., DADA2, QIIME), and determine their implications for reproducibility and interpretation.
5. Support the development of standardised guidelines for future studies by synthesising methodological findings from over 100 research articles.

By focusing on these objectives, this review enhances understanding of how indoor microbiome research is conducted and provides insights to improve the design, harmonisation, and interpretation of future studies in this developing field. Importantly, this work builds upon and extends previous reviews, which have largely focused on describing indoor microbial diversity, identifying dominant taxa, or linking microbial exposure to health outcomes. In contrast, our review systematically examines the methodological foundations of this research. We synthesised and critically evaluated practices across sampling strategies, DNA extraction protocols, sequencing platforms, and bioinformatic pipelines. By demonstrating how methodological variability influences data comparability and interpretation, this review identifies the main obstacles to cross‐study synthesis and proposes directions for the development of standardised and reproducible workflows.

## 2 Methodology

We conducted a systematic literature search covering the period from 2000 to 2025 to identify relevant studies investigating indoor microbial exposure and associated analytical approaches. Although most included studies were published after 2010, we chose to extend the timeframe to the past 25 years, acknowledging that analytical methods and core research themes in this field have remained relatively consistent over time. PubMed was selected as the sole database for this review because it provides comprehensive coverage of biomedical and health‐related studies, is publicly available, and offers a free API (Application Programming Interface), making information more accessible to retrieve using open‐source code (Malinverno et al. 2023). This further aligns closely with our research focus on indoor air quality, asthma‐related outcomes, and microbial communities present in indoor environments. To ensure reproducibility and reduce duplication, we deliberately limited our search to a single platform. Searching across multiple databases can introduce overlapping results and increase the burden of de‐duplication and harmonisation, which complicates downstream screening and data management. We performed an advanced search in PubMed, using Boolean operators (AND/OR) to combine terms related to indoor microbial environments and respiratory health. Key terms included indoor air, dust microbiome, house dust, and built environment, paired with health‐related terms such as asthma, allergy, mycobiome, fungi, and microbial exposure. All search terms were applied to the Title/Abstract fields to ensure relevance. The initial search retrieved 5880 results. To refine this list, we used filters to restrict the results to free full‐text studies published between 2000 and 2025, written in English, and involving human subjects across all age groups. These filters reduced the number of publications to 777. All papers were exported and stored in a shared Zotero library, which served as the central repository for managing references during the review process. The list of documents was then distributed among the research team members, who manually screened each full text and its Supporting Information for eligibility. In cases of disagreement regarding inclusion, team members discussed and justified their decisions until consensus was reached. Studies were included if they investigated indoor environments (e.g., homes, bedrooms, schools, or offices), reported data on microbial communities in indoor air or dust, described at least basic methodological details related to sampling or microbial analysis, and included or discussed health‐related outcomes such as asthma, allergy, or other respiratory indicators. Studies were excluded if they focused exclusively on outdoor environments, lacked microbial data, or did not provide sufficient methodological transparency. Papers that did not reference health impacts or implications, either observed or inferred, were also deprioritized. From the final set of included studies, we extracted a broad set of variables into a structured Table S1 to support comparative analysis. These included metadata such as author, year, study design, population characteristics, and study location, as well as environmental and methodological details, including type of indoor space, sampling site and matrix, sampling method (active or passive), and DNA extraction technique. The decision was made to include information from studies investigating occupational settings, as well. Occupational indoor environments, such as farms and composting facilities, may differ from residential settings in microbial composition and exposure profiles. However, the general principles of sampling and analysis discussed here are broadly applicable across all indoor environments.

**FIGURE 1:**
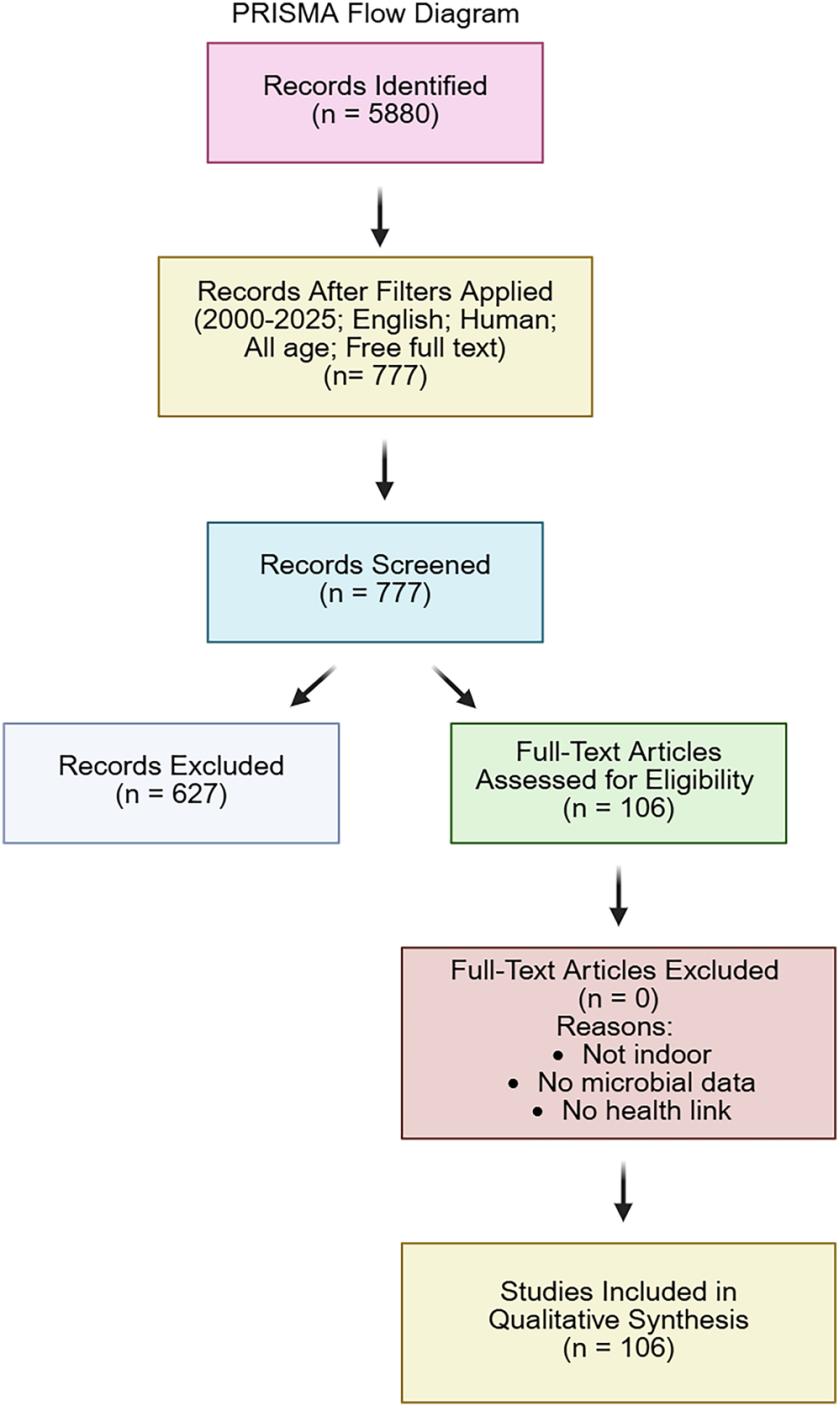
PRISMA flow diagram outlining the study selection process for articles included in this review.

We also recorded sequencing platforms, gene targets (e.g., 16S rRNA gene regions), and bioinformatic pipelines (e.g., QIIME2 [Quantitative Insights into Microbial Ecology], USEARCH DADA2) used for sequence analysis. Where available, we documented the presence of positive and negative controls, microbial normalisation approaches, and the types of microorganisms studied (bacteria, fungi, viruses). Additional extracted data included microbial taxonomic composition, ecological diversity metrics, correlations with environmental factors, and reviewer comments on study‐specific limitations or observations. The full data extraction structure is available in the Table S1. All data extraction fields and reviewer decisions were standardised to promote consistency across the research team. A visual overview of the search and screening process is provided in the PRISMA flow diagram (Figure 1). A total of 106 studies met the inclusion criteria and were included in the final analysis. The main limitation of our search strategy is that by relying solely on PubMed, we may have inadvertently excluded relevant studies indexed in other disciplines such as environmental science, indoor air engineering, or building microbiology. Additionally, we prioritised openly accessible full texts, which may have led to the exclusion of paywalled studies that were otherwise relevant to our research objectives.

## 3 Results

### 3.1 Study Characteristics

In this study, 106 publications from the last 25 years (2000–2025) were selected for analysis. A comprehensive summary of their characteristics is provided in Table S1 in the Supporting Information, with key information presented in Table 1. The included studies were categorised into four groups based on their study design, objectives, and the extent to which health outcomes were assessed. Ecological studies (*n* = 50) focused on describing microbial diversity in dust or air across different indoor environments, without linking the findings to individual health data. Cross‐sectional studies linking microbiome to asthma or allergic outcomes (*n* = 26) included surveys or sampling in populations with reported symptoms, often using questionnaires. Longitudinal studies (*n* = 12) followed individuals or environments over time to assess how microbial exposure affected respiratory health. Lastly, cohort and case–control studies (*n* = 18) specifically selected individuals based on their health status (e.g., asthma diagnosis) to investigate associations with the indoor microbiome, often naming a specific cohort or study population. These designs enabled the inclusion of individual‐level clinical or questionnaire data, allowing for a more robust examination of the links between microbial exposures and health outcomes.

**TABLE 1:** Overview of the studies included in the analysis.

Study type and focus	Targeted organisms with gene regions	Country^a^	Setting	Matrix	Papers
Ecological studies on microbial diversity in dust	Bacteria (16S V3‐V4, V4), Fungi (ITS1)	AU, CN, HR, DK, EE, FI, FR, DE, HK, IS, KR, MY, NO, PK, PT, ES, SE, GB, US	Home, school, university, hospital, office, retail/commercial space, food/drink venue, transport environment, public facility, hotel, childcare, farm, school environments	Indoor and outdoor air; airborne fungi and microbes; settled, vacuumed, stable, mattress, floor, surface, incubated carpet HVAC, house, living room floors, and road dust; filters; greenbelt soil; saliva (mouthwashes); skin surface swabs; and surfaces (e.g., bed headboard, blanket, fridge door seal, kitchen ventilator, remote control, shower curtain, toilet flush button, TV screen)	(Guo et al. 2020; Kettleson et al. 2015; Amin et al. 2023; An et al. 2023; Araujo et al. 2008; Asif et al. 2018, 2019; Barberán et al. 2015; Chen et al. 2010, 2024; Coombs et al. 2018; Cox et al. 2017, 2021; Dannemiller, Gent, et al. 2016; Fu, Li, et al. 2020; Fu, Norbäck, et al. 2020; Fu, Ou, et al. 2021b; Hanson et al. 2016; Hassan et al. 2021; Hickman et al. 2022; Hoisington et al. 2014; Jarma et al. 2024; Jo and Seo 2005; Kauserud et al. 2025; Lee and Jo 2006; Lee, Yang, et al. 2021; Leung et al. 2014, 2018; Li et al. 2013, 2022; Maestre et al. 2018; Mäki et al. 2021; Martikainen et al. 2021; Martin‐Sanchez et al. 2021; Noris et al. 2011; Núñez and García 2022; Park et al. 2021, 2022; Sautour et al. 2009; Shan et al. 2020; Šunić et al. 2025; Täubel et al. 2009; Tong et al. 2017; Veillette et al. 2013; Vestergaard et al. 2018; Vidal‐Quist et al. 2021; Weikl et al. 2016; Yang et al. 2022; Zhou et al. 2021)
Cross‐sectional studies linking microbiome to asthma or allergic outcomes	Bacteria (16S V3‐V5, V4), Fungi (ITS2)	AT, BE, CN, HR, DK, FR, DE, IN, IR, IT, JP, MY, NL, NO, PL, KR, ES, SE, CH, TW, TH, TR, AE, GB, US	Homes, schools, dormitory	Settled, vacuum, floor, surface, bed, bedroom floor, indoor, desks, chairs, airborne, and indoor air dust; hypopharyngeal aspirates (infant airway samples)	(Gupta et al. 2020; Ege et al. 2011, 2012; Dannemiller, Gent, et al. 2016; Birzele et al. 2017; Celtik et al. 2011; Ciaccio et al. 2014, 2015; Cochran et al. 2022; Eiffert et al. 2016; Fu, Ou, et al. 2021a; Fu, Yuan, et al. 2021; Fu, Li, et al. 2021; Fu et al. 2024; Isa et al. 2022; Lee, Wyss, et al. 2021; Lee et al. 2024; Ludwig et al. 2017; Onwusereaka et al. 2024; Richardson et al. 2019; Rittenour et al. 2014; Sun et al. 2022; Šarac et al. 2025; Yamamoto et al. 2011; Leung et al. 2008)
Longitudinal studies on microbial exposure and respiratory outcomes	Bacteria (16S), Fungi (ITS1, ITS2)	CN, FI, DE, NL, NO, US	Homes, kindergartens, dormitory	Settled, floor, doorframe, bedding and carpet dust; air samples (indoors and in the yard); doormat debris (dirt, airborne dust, plant fragments, and animal/human material)	(Cox et al. 2022; Tischer et al. 2016; Chew et al. 2001, 2003; Estensmo et al. 2021, 2022; Hui et al. 2019; Jayaprakash et al. 2017; Nygaard and Charnock 2018; Ross et al. 2000; Sitarik et al. 2018; Zhao et al. 2025)
Cohort and case–control studies on microbiome and health	Bacteria (16S), Fungi (ITS)	AT, CA, CN, EE, FI, FR, DE, IS, IR, SG, ES, SE, CH, GB, US	Homes, farm homes	Settled, vacuumed, floor, bed, windowsills, living room, sofa, play areas, and sleeping surface dust; stool; indoor and outdoor air samples; saliva; sputum; skin; nasal mucosa	(Vandenborght et al. 2021; Dalton et al. 2024; Kirjavainen et al. 2019; Karvonen et al. 2019; Böttcher et al. 2003; Ding et al. 2020; Fairs et al. 2013; Fujimura et al. 2012; Konya et al. 2014; Loo et al. 2018; Niemeier‐Walsh et al. 2021; O'Connor et al. 2018; Rocchi et al. 2015; Shabankarehfard et al. 2017; Tang et al. 2024; Valkonen et al. 2015, 2018; Wang et al. 2023)

^a^ Australia (AU), Austria (AT), Belgium (BE), Canada (CA), China (CN), Croatia (HR), Denmark (DK), Estonia (EE), Finland (FI), France (FR), Germany (DE), Hong Kong (HK), Iceland (IS), India (IN), Iran (IR), Italy (IT), Japan (JP), Malaysia (MY), the Netherlands (NL), Norway (NO), Pakistan (PK), Poland (PL), Portugal (PT), Singapore (SG), South Korea (KR), Spain (ES), Sweden (SE), Switzerland (CH), Taiwan (TW), Thailand (TH), Turkey (TR), the United Arab Emirates (UAE), the United Kingdom (GB), and the United States of America (US).

Most studies (*n* = 54) focused on child‐related environments, with homes—including households and houses—being the most frequently investigated setting (*n* = 70), followed by schools (*n* = 14) and kindergartens (*n* = 5). This emphasis reflects the high vulnerability of children to respiratory diseases and the crucial role of early‐life microbial exposures in shaping immune system development. Their immature immune and respiratory systems make them particularly sensitive to environmental and microbial influences. This study design, therefore, provides an opportunity to capture early indicators of disease risk and identify preventive strategies before chronic conditions emerge. Additionally, research involving children is often more feasible from a study design perspective, as their health outcomes can more directly reflect environmental exposures without the confounding effects of pre‐existing conditions. By contrast, in elderly or immunocompromised populations, respiratory outcomes may be strongly influenced by underlying illnesses or treatments, making it more challenging to disentangle the specific contribution of the indoor microbiome. This complexity, combined with recruitment and ethical considerations, likely contributes to the comparatively fewer studies examining these vulnerable groups, despite their heightened susceptibility to indoor air quality and microbial exposures.

In terms of sample type, dust was the predominant matrix, appearing in 77 studies and encompassing various forms such as settled dust, house dust, and floor dust. Air samples were the most used matrix in 29 studies. Geographically, most studies were conducted in the United States (*n* = 35), followed by China (*n* = 16), Finland (*n* = 6), and both Malaysia and Norway (*n* = 5 each).

### 3.2 Asthma and Associated Diseases

Asthma is a prevalent chronic respiratory condition affecting both children and adults worldwide. In the European Union, approximately 6% of the population is affected, with notable variation among countries (EUROSTAT 2021). In the United States, asthma affects about 8% of the population, including roughly 7% of children (Park et al. 2022; Global Initiative for Asthma 2015; Centers for Disease Control and Prevention 2019). Asthma frequently coexists with other respiratory conditions such as rhinitis and wheezing, and its prevalence is influenced by a complex interplay of environmental, microbial, and socio‐economic factors (Cox et al. 2022).

Indoor environmental exposures, particularly dampness, mould, and biological agents such as bacteria, fungi, and allergens, have been linked to increased asthma risk and severity (WHO 2009; Stocka et al. 2024; Nastasi et al. 2020). Fungal genera such as *Cladosporium* and *Aspergillus* are often associated with asthma exacerbations in homes with significant fungal contamination (Cox et al. 2022), while exposure to indoor dust and mould can significantly increase the risk of asthma and allergic rhinitis (Nastasi et al. 2020). Conversely, certain microbial exposures, including those encountered in farm environments or associated with cockroaches, mice, and cats, may have a protective effect, supporting immune system regulation and reducing asthma risk (Topalušić et al. 2022; Martikainen et al. 2021; O'Connor et al. 2018). Socio‐economic conditions also modulate disease prevalence, with disadvantaged urban areas experiencing higher asthma rates and greater exposure to poor indoor air quality and mould (Eiffert et al. 2016). In summary, asthma is shaped by a combination of microbial, environmental, and socio‐economic factors, with indoor exposures playing a significant role in both disease risk and protection (Fu, Norbäck, et al. 2020; Fu, Ou, et al. 2021a, 2021b; Fu, Li, et al. 2021).

### 3.3 Sampling and Isolation Methods

#### 3.3.1 Sampling

Out of the 106 papers reviewed, 18 used active sampling methods (e.g., air sampling with various devices) as a standalone approach, while 66 relied solely on passive sampling methods, as shown in Table 2. Eight studies combined active and passive sampling, five studies used passive sampling along with biological samples (nasal or skin swabs), and one study used both active and passive methods along with biological sample collection (sputum). Seven studies included human sampling of biomonitoring indicators for asthma and other negative health outcomes.

**TABLE 2:** Sampling methods employed in the retrieved studies.

Sampling methods	Number of papers	References
Active	18	(Araujo et al. 2008; Asif et al. 2018, 2019; Chen et al. 2024; Hassan et al. 2021; Jo and Seo 2005; Lee and Jo 2006; Lee, Yang, et al. 2021; Leung et al. 2014, 2018; Núñez and García 2022; Sautour et al. 2009; Vestergaard et al. 2018; Yang et al. 2022; Zhou et al. 2021; Richardson et al. 2019; Chew et al. 2003; Zhao et al. 2025)
Passive	66	(Vandenborght et al. 2021; Cox et al. 2022, 2021; Dalton et al. 2024; Ege et al. 2011, 2012; Kettleson et al. 2015; Kirjavainen et al. 2019; Tischer et al. 2016; Karvonen et al. 2019; Amin et al. 2023; An et al. 2023; Barberán et al. 2015; Chen et al. 2010; Dannemiller, Gent, et al. 2016; Fu, Li, et al. 2020, 2021; Fu, Norbäck, et al. 2020, 2021; Fu, Ou, et al. 2021b, 2021a; Hickman et al. 2022; Jarma et al. 2024; Kauserud et al. 2025; Li et al. 2022; Mäki et al. 2021; Martikainen et al. 2021; Martin‐Sanchez et al. 2021; Park et al. 2021, 2022; Shan et al. 2020; Šunić et al. 2025; Veillette et al. 2013; Vidal‐Quist et al. 2021; Weikl et al. 2016; Celtik et al. 2011; Ciaccio et al. 2014, 2015; Cochran et al. 2022; Eiffert et al. 2016; Fu, Yuan, et al. 2021; Fu et al. 2024; Isa et al. 2022; Lee, Wyss, et al. 2021; Lee et al. 2024; Ludwig et al. 2017; Onwusereaka et al. 2024; Sun et al. 2022; Yamamoto et al. 2011; Leung et al. 2008; Chew et al. 2001; Estensmo et al. 2021; Jayaprakash et al. 2017; Nygaard and Charnock 2018; Sitarik et al. 2018; Böttcher et al. 2003; Ding et al. 2020; Fujimura et al. 2012; Loo et al. 2018; O'Connor et al. 2018; Rocchi et al. 2015; Valkonen et al. 2015, 2018; Wang et al. 2023; Nastasi et al. 2020; Dannemiller, Leaderer, and Peccia 2016)
Active and passive	8	(Coombs et al. 2018; Cox et al. 2017; Hanson et al. 2016; Tong et al. 2017; Rittenour et al. 2014; Estensmo et al. 2021; Ross et al. 2000; Shabankarehfard et al. 2017)
Passive and biological samples	5	(Gupta et al. 2020; Täubel et al. 2009; Birzele et al. 2017; Niemeier‐Walsh et al. 2021; Tang et al. 2024)
Active, passive and biological samples	1	(Fairs et al. 2013)
Material recovery	2	(Li et al. 2013; Hui et al. 2019)
Material recovery and passive	2	(Maestre et al. 2018; Konya et al. 2014)
Material recovery, active and passive	2	(Hoisington et al. 2014; Noris et al. 2011)
Material recovery, active, passive and biological samples	1	(Guo et al. 2020)

Two studies focused exclusively on material recovery (e.g., HVAC filters and doormats), and two other studies combined material recovery with passive sampling. Another two used all three environmental sampling methods (active, passive, and material recovery), whereas one study employed all three sampling methods (i.e., active, passive, and material recovery) and investigated biological samples (mouthwashes).

The most used passive sampling methods were vacuuming (66 studies), followed by swabs (13 studies), and electrostatic dust collectors (EDCs) (6 studies). There is a growing trend toward the use of passive sampling methods, primarily due to their low cost and ease of use. Analysing settled dust from indoor environments has become a commonly adopted approach for evaluating microbial contamination of indoor air in environmental studies (Leppänen et al. 2018; Park et al. 2018; Viegas et al. 2021). This type of dust has also been used to detect contamination by mycotoxins (Viegas et al. 2018). Moreover, settled dust provides a supportive environment for bacterial growth, and is therefore regarded as a potential reservoir of bacterial contamination (Bouillard et al. 2005).

Dust that passively accumulates on indoor surfaces can be collected using vacuum‐based methods, often targeting carpets, furniture, or flooring, and captured in filters, tubes, or nylon sampling socks (Leppänen et al. 2014). The microbial content in floor dust may originate from several sources, including outdoor air infiltration, human occupants, pets, pests, or microbial growth within building materials and furnishings (Dunn et al. 2013). Settled dust is particularly useful as a proxy for inhalation exposure because it reflects longer‐term contamination and is less affected by short‐term changes in ventilation or indoor activity (Meyer et al. 2004). Its significance also lies in the potential for dust‐associated microorganisms and their by‐products to become airborne again, increasing the risk of inhalation exposure (Aleksic et al. 2017). It should be noted, however, that this sampling method is limited to microorganisms capable of surviving in a dry environment, primarily in the form of resistant spores, as discussed in more detail in Section 3.3.2. Isolation of genetic material.

Electrostatic dust cloths (EDCs) have emerged as a widely used passive sampling tool, as demonstrated by Adams et al. (2021) and Viegas et al. (2022). These cloths are particularly effective for long‐term sampling, depending on the expected contamination levels, and are compatible with various analytical techniques following a simple extraction procedure (Viegas et al. 2022). EDCs have been utilised to monitor a wide array of microorganisms, including fungi, bacteria, and viruses (Furst et al. 2025, 2024; Dias et al. 2024; Viegas et al. 2021, 2022; Sequeira et al. 2024), as well as microbial by‐products like endotoxins and mycotoxins (Kristono et al. 2019; Viegas et al. 2024). Their versatility has made them valuable for environmental monitoring across a range of indoor and occupational settings.

A wide range of sampling sites was used in the studies analysed. Floors were the most frequently sampled, appearing in 52 studies and representing the largest proportion of total samples. Active sampling methods (air samples) were employed in 30 studies, while 13 studies used passive sampling techniques (surface swabs), targeting areas such as carpets (seven studies), door frames (six studies), and various other surfaces, including curtains and furniture. Some studies also analysed fewer common matrices: mattresses were sampled in 18 studies, while HVAC components, such as return air grilles and air conditioning filters, were included in only two studies. For air sampling, a variety of devices were used, including the single‐stage Anderson sampler, Inspirotec sampler, Gilian 5000, and QuickTake 30 sample pump.

It is well established that sampling campaigns aimed at assessing microbial contamination should incorporate multiple sampling methods (Cervantes et al. 2025; Dias et al. 2024). The heterogeneity of DNA extraction methods introduces systematic biases that influence microbial diversity outcomes across studies. Differences in isolation methods and chemicals used can affect cell lysis efficiency, inhibitor removal, as well as the DNA yield and therefore influence the result of DNA amplification and the recovery of low‐abundance microorganisms, thereby influencing observed alpha‐ and beta‐diversity (Amin et al. 2023). As a result, cross‐study comparisons of indoor microbiomes must be interpreted cautiously, since observed compositional differences may partly arise from methodological rather than environmental variation.

#### 3.3.2 Isolation of Genetic Material

Isolation of genetic material is a crucial step in sample preparation, particularly for ultra‐low biomass matrices such as air and dust. Many microorganisms in bioaerosols, including bacteria and fungi, persist as resilient spores that protect their genomic content. These spores require harsh lysis conditions to effectively break cell walls, ensuring comprehensive microbial recovery and minimising bias in downstream analyses. Thus, robust and efficient DNA extraction protocols are essential to maximise yield without compromising the integrity of the genetic material, which is vital for accurately characterising indoor microbiomes. Our systematic analysis of 106 studies revealed key trends and preferences in DNA isolation methods for characterising the indoor microbiome. In the early 2000s, 13 studies on indoor microbiomes relied primarily on culturomics with microbial load reported as CFU/m3 (Jo and Seo 2005; Lee and Jo 2006; Chew et al. 2003; Ross et al. 2000). However, with the advent and increased accessibility of NGS technologies, culture‐independent methods became more prevalent. Two main strategies emerged: DNA extraction from cultured colonies and direct extraction of DNA from collected air or dust samples. The extraction of genetic material from accumulated cultured biomass is relatively straightforward, with several commercially available kits providing reliable performance (Asif et al. 2018, 2019; Hassan et al. 2021; Li et al. 2013; Sautour et al. 2009).

In contrast, extracting DNA directly from air and dust samples poses a greater challenge due to the typically low microbial load, the presence of inhibitors, and the complexity of the sample matrix. Chemical lysis, often supplemented with enzymatic treatment, was commonly employed to facilitate the breakdown of microbial cells (Ege et al. 2011; Hoisington et al. 2014; Noris et al. 2011). However, commercial column‐based kits designed for complex environmental matrices (e.g., soil, water, plant tissues) became the most widely used approach. Among these, the PowerSoil DNA Isolation Kit (Qiagen, Germany) was the preferred option appearing in 30 studies, followed by the FastDNA SPIN Kit for Soil (MP Biomedicals, USA) used in eight studies, while the High Pure PCR Template kit (Roche, Germany) was used in six studies.

### 3.4 Data Analysis and Sequencing

#### 3.4.1 Data Analysis

**FIGURE 2:**
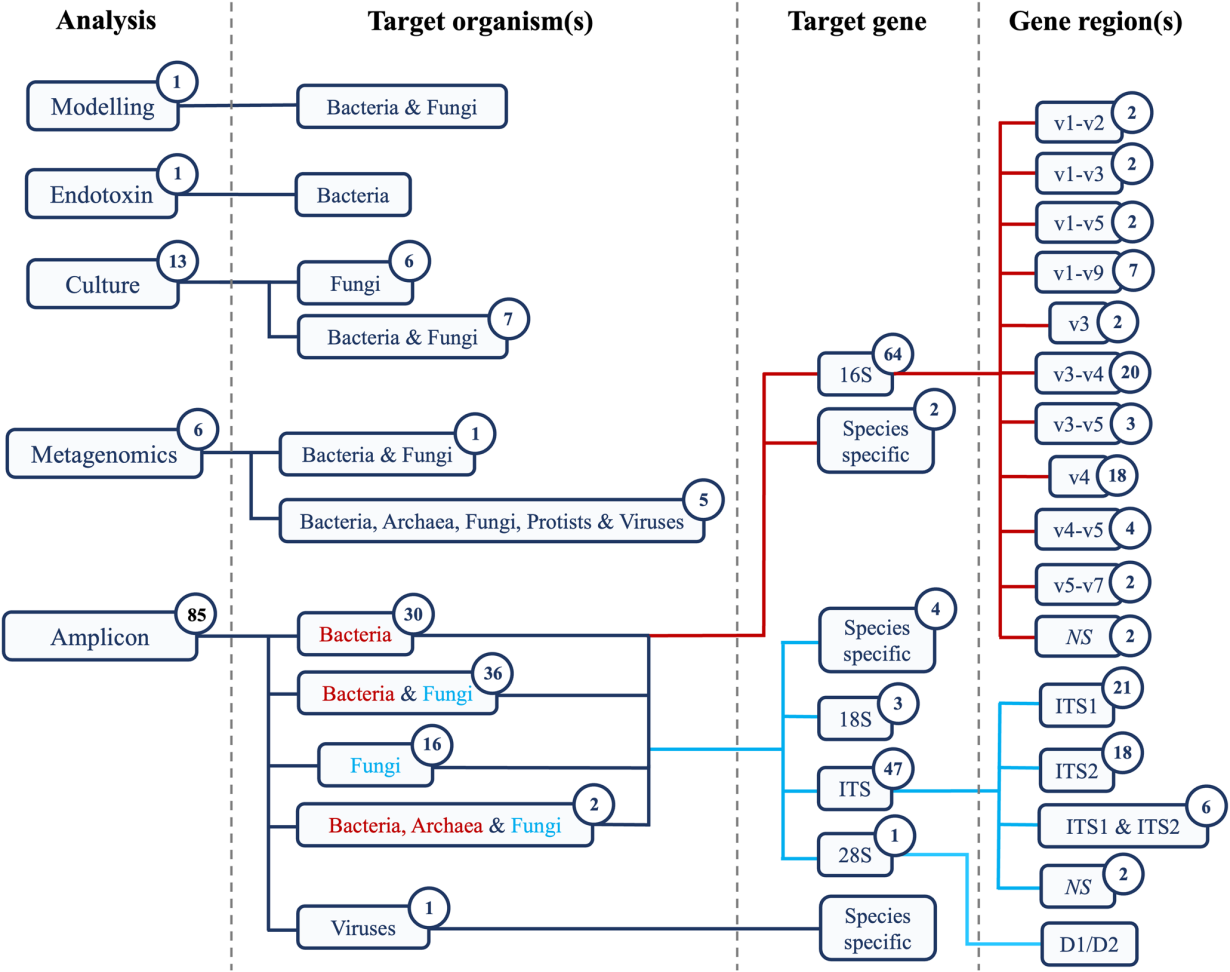
A summary of the types of analyses conducted in the extracted studies (*n* = 106). Circles represent the number of studies in each category. Here, ‘Amplicon’ refers to any amplicon‐based analysis, not just amplicon sequencing. For these particular studies, the target microorganism(s), as well as the specific genes and regions, are detailed. Some studies targeted multiple bacterial and/or fungal genes, meaning ‘Target gene’ numbers exceed the number of ‘Target organism(s)’. Studies that included one or more species‐specific primers for a given target organism, for example, bacteria, are grouped. NS = methods not stated.

A variety of analyses, including computational (1/106), endotoxin (1/106), culture‐based (13/106), and molecular (91/106), were implemented across the included publications (Figure 2). In one case, modelling was used to demonstrate the protective effect of farm home dust microbiota against childhood asthma (Kirjavainen et al. 2019). In another study, endotoxin concentrations were quantified in dust taken from homes in Estonia and Sweden (Böttcher et al. 2003). A total of 13 studies adopted a culture‐based approach. Moreover, around half of these studies focused solely on fungal contamination, whilst the rest included both fungi and bacteria (Figure 2).

However, a molecular approach was the most common, with 91 out of the 106 studies opting to use molecular techniques, the majority of which were amplicon‐based (Figure 2). Quantitative polymerase chain reaction (qPCR) was used in nine of the studies to determine absolute microbial abundances, whilst the other 76 studies used amplicon‐based methods to characterise microbial communities. Although amplicon sequencing was the preferred method in 74 of these studies, one study employed microarrays, and another used restriction fragment length polymorphism (RFLP) analysis. Lastly, a total of 6 studies carried out metagenomic shotgun sequencing as an alternative to amplicon‐based community analysis.

#### 3.4.2 Sequencing Strategies and Targets

A total of 75 studies implemented amplicon sequencing for microbial identification and community analysis. The 16S rRNA gene was the primary target for bacterial profiling, with most studies targeting the V3–V4 and V4 regions. Full‐length 16S rRNA sequencing (V1–V9) was also implemented (Figure 2). To assess fungal diversity, several rRNA regions were targeted, including the 18S small subunit, the 28S large subunit, and the internal transcribed spacer regions 1 (ITS1) and (ITS2) that separate these genes. Of these, the ITS region was the most targeted, with the studies showing a near equal split between ITS1 and ITS2. Notably, 36 studies decided on a dual‐target approach, sequencing both bacterial and fungal genes. Moreover, 2 studies targeted archaeal genes in addition to the aforementioned genes. Only a single study targeted a virus, in which species‐specific primers were used. Similarly, species‐specific primers were also implemented in several fungal and bacterial studies (Figure 2). The lack of standardisation in the choice of hypervariable regions within ribosomal subunits is rooted in discrepancies in sequence read lengths across NGS platforms. For example, the Illumina NextSeq is limited to 2 × 150 bp, which allows sequencing of either the V3 or V4 regions individually. The Illumina MiSeq, which supports up to 2 × 300 bp, is commonly used to sequence the combined V3–V4 region (~460 bp). Only long‐read sequencing platforms, such as Pacific Biosciences (PacBio) and Oxford Nanopore Technologies (ONT), can sequence near full‐length 16S rRNA gene amplicons in high throughput.

Less than 10% of molecular studies employed metagenomic shotgun sequencing as a means of assessing microbial diversity. Although initially untargeted, metagenomic sequencing data are generally analysed with specific group(s) of organisms in mind. Out of the 6 metagenomic studies, bacterial and fungal communities were the focus of one, whereas the other 5 studies were broader and included sequencing data for archaea, protists, and viruses, as well as bacteria and fungi.

#### 3.4.3 Sequencing Platforms and Analytical Tools

**FIGURE 3:**
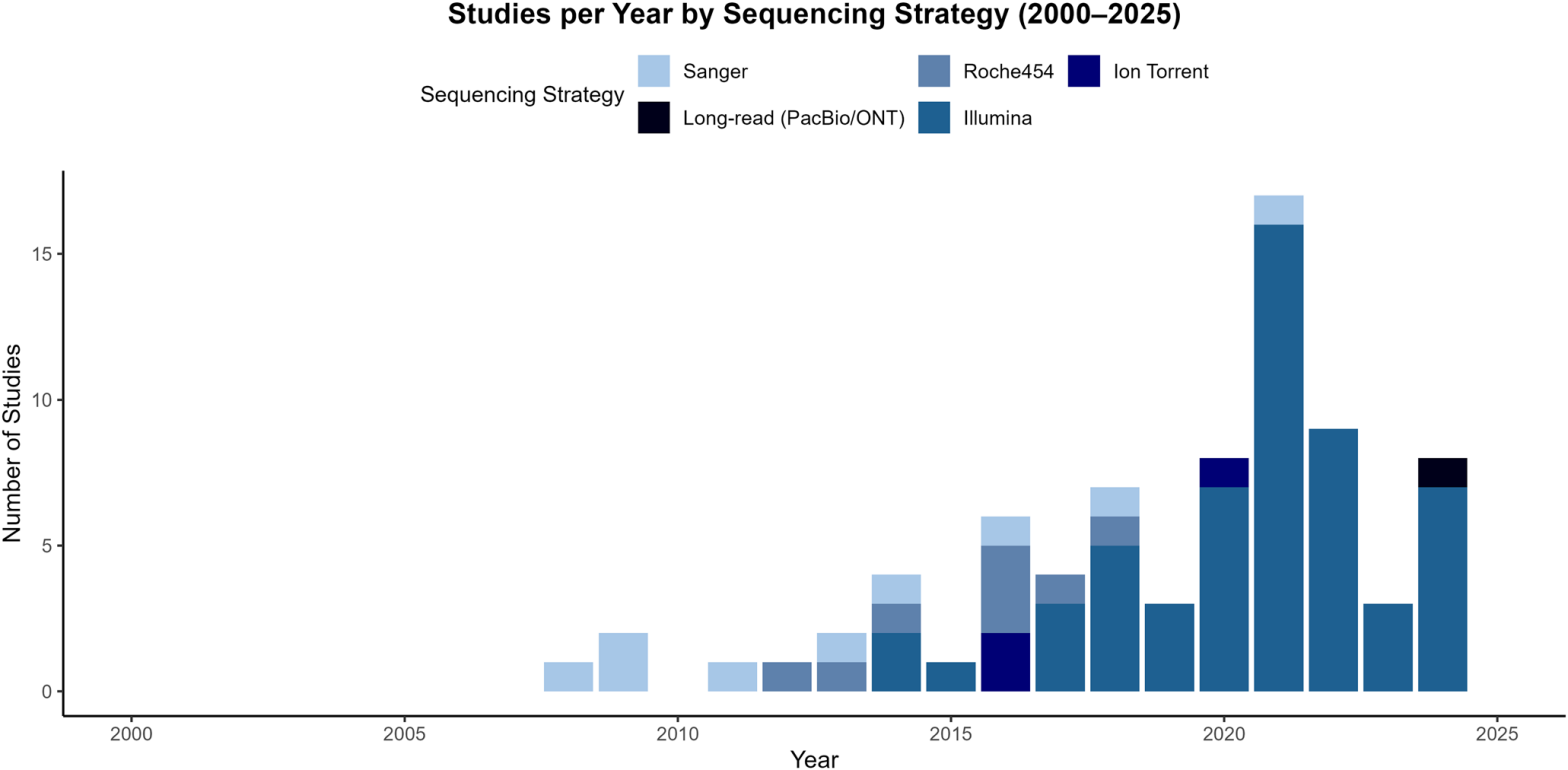
Temporal trends in sequencing strategies used in indoor microbiome studies from 2000 to 2025. The figure shows a shift from early Sanger and Roche 454 methods to the dominance of Illumina platforms, with recent adoption of Ion Torrent and long‐read technologies (PacBio and ONT).

Based on our review of 106 publications, 79 studies utilised sequence‐based approaches. Next‐generation sequencing (NGS) has become the standard approach for characterising microbial communities in dust, air, and other indoor matrices. Illumina platforms, particularly MiSeq and HiSeq, dominate the field due to their high throughput, accuracy, and cost‐effectiveness for amplicon‐based sequencing. Among studies, over 90% utilised Illumina systems (Figure 3). Sanger sequencing, once the standard for microbial identification, has largely been replaced by these newer technologies because of its low throughput and higher per‐sample cost (Table 3).

**TABLE 3:** Overview of sequencing strategies and platform usage in indoor microbiome studies.

Common sequencing strategy	Method	Platform examples	Number of publications	Publications
1. Sanger sequencing		ABI 3730/3730xl Capillary Sequencer	9	(Asif et al. 2018; Noris et al. 2011; Sautour et al. 2009; Täubel et al. 2009; Vidal‐Quist et al. 2021; Weikl et al. 2016; Rittenour et al. 2014; Leung et al. 2008; Fairs et al. 2013)
2. Next‐generation sequencing (NGS)	Pyrosequencing (~2005–2015)	Roche 454 GS FLX	9	(Kettleson et al. 2015; Dannemiller, Gent, et al. 2016; Hanson et al. 2016; Hoisington et al. 2014; Li et al. 2013; Birzele et al. 2017; Nygaard and Charnock 2018; Fujimura et al. 2012; Dannemiller, Leaderer, and Peccia 2016)
	Semiconductor Sequencing (~2010—present)	Ion Torrent PGM/Ion S5/Ion S5 XL	3	(Dannemiller, Gent, et al. 2016; Shan et al. 2020; Dannemiller, Leaderer, and Peccia 2016)
	Short‐read NGS (~2010—present)	Illumina	59	(Vandenborght et al. 2021; Gupta et al. 2020; Cox et al. 2022, 2021; Dalton et al. 2024; Guo et al. 2020; Karvonen et al. 2019; Amin et al. 2023; An et al. 2023; Barberán et al. 2015; Chen et al. 2024; Coombs et al. 2018; Fu, Li, et al. 2020, 2021; Fu, Norbäck, et al. 2020, 2021; Fu, Ou, et al. 2021a, 2021b; Hickman et al. 2022; Jarma et al. 2024; Kauserud et al. 2025; Leung et al. 2014, 2018; Li et al. 2022; Maestre et al. 2018; Mäki et al. 2021; Martikainen et al. 2021; Martin‐Sanchez et al. 2021; Núñez and García 2022; Park et al. 2021, 2022; Šunić et al. 2025; Tong et al. 2017; Vestergaard et al. 2018; Yang et al. 2022; Zhou et al. 2021; Cochran et al. 2022; Fu, Yuan, et al. 2021; Fu et al. 2024, 2022; Isa et al. 2022; Lee et al. 2024; Onwusereaka et al. 2024; Richardson et al. 2019; Sun et al. 2022; Šarac et al. 2025; Estensmo et al. 2021, 2022; Hui et al. 2019; Jayaprakash et al. 2017; Sitarik et al. 2018; Ding et al. 2020; Konya et al. 2014; Loo et al. 2018; Niemeier‐Walsh et al. 2021; Tang et al. 2024; Wang et al. 2023; Nastasi et al. 2020)
3. Third generation/Long‐read Sequencing	PacBio		1	(Tang et al. 2024)
	Nanopore	ONT	0	

In recent years, third‐generation sequencing technologies such as PacBio and Oxford Nanopore Technologies (ONT) have begun to emerge in microbiome research, though they remain less commonly used. These long‐read platforms offer significant advantages over short‐read sequencing by enabling near full‐length 16S rRNA or ITS reads that improve taxonomic resolution, phylogenetic accuracy, and the detection of complex or repetitive genomic regions. PacBio provides highly accurate consensus sequences (up to Q50) but remains relatively costly, whereas ONT offers a more affordable alternative benefitting from recent innovation such as R10.4.1 flow cells, V14 chemistry, and duplex sequencing. Our recent work further demonstrated that these advances have made ONT compatible with ultra‐low biomass air samples: although performed on outdoor air, the approach enabled effective profiling of airborne bacterial communities as well as the distribution of antimicrobial resistance genes, underscoring the potential applicability of long‐read sequencing to indoor air microbiome studies as technological sensitivity continues to improve (Reska et al. 2024). Preliminary indoor studies support this potential; for example, ONT near full‐length 16S rRNA sequencing of building‐dust microbiomes provided substantially greater species‐level resolution than short‐read sequencing, revealing numerous taxa not detected by Illumina (Nygaard et al. 2020). Advances in ONT library preparation and PCR optimization have shown that high G + C content bacterial genomes can be reconstructed with quality comparable to PacBio, enabling near‐complete genome assemblies using ONT alone (Soto‐Serrano et al. 2024). Advances in long‐read assembly and polishing tools have further improved genome quality, enabling more complete reconstruction of complex microbial genomes from mixed environmental samples. Additionally, ONT now supports accurate microbial epigenetic profiling through improved methylation calling tools (e.g., Nanomotif and MicrobeMod), extending capabilities once unique to PacBio. Although most large‐scale applications have focused on high‐biomass environments such as soil and sediment samples where ONT revealed 15,314 previously undescribed microbial species across 1086 new genera, expanding known prokaryotic diversity by 8% (Sereika et al. 2025). Continued gains in accuracy and sensitivity highlight the strong potential of long‐read platforms for future indoor and airborne microbiome studies.

#### 3.4.4 Bioinformatic Pipelines and Quality Control

Bioinformatic processing of sequencing data commonly involves widely adopted open‐source pipelines. Raw data processing from FASTQ files, including quality filtering, chimera removal, sequence clustering or denoising, and taxonomic assignment, is commonly performed using pipelines such as QIIME2 (Bolyen et al. 2019), DADA2 (Callahan et al. 2016), USEARCH/VSEARCH (Callahan et al. 2016; Rognes et al. 2016), and mothur (Schloss et al. 2009), with newer approaches such as LACA (Hui et al. 2025), emerging to support more advanced analysis. Reference databases including SILVA (Quast et al. 2013), Greengenes2 (McDonald et al. 2024), RDP (Cole et al. 2014), and UNITE (Nilsson et al. 2019) are used for bacterial and fungal taxonomic classification, depending on the target domain and marker gene. Downstream analyses, such as diversity estimation and community structure profiling, are typically conducted in QIIME2 or Phyloseq (McMurdie and Holmes 2013). Functional prediction tools (e.g., PICRUSt, HUMAnN2) and ecological guild classifiers such as FUNGuild are also used to infer potential microbial functions and ecological roles.

Across evaluated studies, QIIME/QIIME2 was used most frequently (19 studies), followed by DADA2 (10 studies), whereas mothur was rarely used (three studies). However, comparison across studies is complicated by heterogeneity within pipelines themselves. For instance, some studies generated ASVs using DADA2 with QIIME2 while others employed OTU clustering with USEARCH. Such differences in feature generation create challenges for meta‐analysis, as alpha‐diversity estimates and taxonomic resolution vary depending on whether OTUs or ASVs are used and on clustering thresholds (commonly 97% or 100%). The growing implementation of shotgun metagenomic sequencing further adds to the diversity of analytical approaches and complicates direct comparisons across studies. Most widely used pipelines were primarily developed for paired‐end and/or short‐read data (mainly Illumina), and many steps are not directly applicable to long‐read platforms such as ONT and PacBio, where reads commonly exceed 1 kb. Although new methods—such as the LACA pipeline (Hui et al. 2025)—are emerging to better accommodate long‐read processing and classification, their adoption in indoor microbiome research remains limited.

A further concern we identified is the inconsistent reporting of quality control measures and data availability. While many studies include negative controls (e.g., blanks), positive controls such as mock communities are less common. Among sequencing studies analysed, 39% did not report any controls, 33% used negative controls only, 4% used positive controls, and just 16% included both. Clear definitions are also needed, as “negative control” may refer to an extraction blank or a PCR no‐template control (NTC), yet best practice would include both.

Data sharing practices are similarly inconsistent. Despite long‐established norms and funder requirements, 48% of the studies did not make sequencing data publicly available. An additional 5% provided only processed data while 39% deposited raw data in public repositories such as the Sequence Read Archive (SRA). Encouragingly, use of platforms like QIITA (7% of studies), which support standardised sharing of raw data, metadata and analysis workflows, may help reduce barriers to cross‐study comparability and reproducibility.

### 3.5 Ecological and Microbial Findings

#### 3.5.1 Dust as a Reservoir of Microbiota

The composition of indoor microbiomes has been widely characterised, with numerous bacterial and fungal genera frequently detected in household dust. Bacterial taxa commonly identified across indoor studies include *Staphylococcus*, *Streptococcus*, *Corynebacterium*, *Micrococcus*, and *Bacillus*, which are often derived from human skin, oral, and respiratory microbiota (Ege et al. 2011; Asif et al. 2019; Barberán et al. 2015; Chen et al. 2010; Hanson et al. 2016; Martikainen et al. 2021; Veillette et al. 2013; Ege et al. 2012; Fu et al. 2024; Lee, Wyss, et al. 2021; Onwusereaka et al. 2024; Šarac et al. 2025; Hui et al. 2019; Jayaprakash et al. 2017). These taxa dominate in environments with high human occupancy and limited ventilation or with frequent surface contact by residents. Environmental bacteria, including *Sphingomonas*, *Ralstonia*, *Pseudomonas*, and *Burkholderia*, are frequently reported as well, particularly in homes with more natural ventilation or proximity to vegetation (Gupta et al. 2020; Fu, Li, et al. 2020; Hoisington et al. 2014; Leung et al. 2014; Li et al. 2013; Park et al. 2021; Sun et al. 2022). Multiple studies, including those by Cox et al. (2022, 2017, 2021), Dannemiller, Gent, et al. (2016); Dannemiller, Leaderer, and Peccia (2016), and Fu, Li, et al. (2020); Fu, Norbäck, et al. (2020); Fu et al. (2021a); Fu et al. (2024), have found that indoor dust often contains microbiomes from outdoor environments. Additionally, the composition of indoor microbial communities is shaped by building characteristics such as moisture, ventilation, and occupant behaviour (Cox et al. 2022, 2017, 2021; Dannemiller, Gent, et al. 2016; Fu, Li, et al. 2020; Fu, Norbäck, et al. 2020; Leung et al. 2014; Park et al. 2021; Fu et al. 2024, 2022).

Fungal communities in indoor environments are diverse and are often dominated by globally prevalent genera such as *Cladosporium*, *Aspergillus*, *Penicillium*, *Alternaria*, and *Wallemia*, with spores originating from both outdoor air and indoor surfaces (Dannemiller, Gent, et al. 2016; Sautour et al. 2009; Šunić et al. 2025; Weikl et al. 2016; Isa et al. 2022; Rittenour et al. 2014; Chew et al. 2001; Estensmo et al. 2021; Rocchi et al. 2015; Dannemiller, Leaderer, and Peccia 2016; Fairs et al. 2010). Advanced sequencing‐based studies have expanded the known diversity of indoor fungi, revealing frequent detection of *Epicoccum*, *Aureobasidium*, and *Didymella* species (Vandenborght et al. 2021; Isa et al. 2022; Sun et al. 2022; Estensmo et al. 2021). *Aspergillus* and *Penicillium* are commonly present in indoor air, but their concentrations are significantly higher in damp or water‐damaged buildings (Tischer et al. 2016; Eiffert et al. 2016; Richardson et al. 2019; Estensmo et al. 2022; Jayaprakash et al. 2017), and their presence has been linked to allergic disease and asthma exacerbation (Karvonen et al. 2019; Shan et al. 2020; Richardson et al. 2019).

While the abundance of specific taxa can vary, their repeated detection suggests stable persistence in indoor environments. The concept of a “core indoor microbiome” is often invoked to describe the consistently occurring microbial taxa found across built environments; defining such a universal core remains methodologically and ecologically unfeasible. Indoor microbial communities are highly context‐dependent, shaped by diverse factors including building design, ventilation type, occupant density, cleaning practices, material composition, and local climate. Because most indoor microorganisms derive from transient sources such as humans, pets, and outdoor air (Kettleson et al. 2015; Cox et al. 2021; Leung et al. 2014; Maestre et al. 2018; Mäki et al. 2021; Šunić et al. 2025; Täubel et al. 2009; Šarac et al. 2025; Jayaprakash et al. 2017; Sitarik et al. 2018; Loo et al. 2018; Dannemiller, Leaderer, and Peccia 2016), rather than from an intrinsic indoor ecosystem, their composition reflects environmental inputs rather than shared endemic taxa. Furthermore, variations in sampling strategy, sequencing methodology, and bioinformatic processing generate inconsistent results across studies.

#### 3.5.2 Ecological Characteristics and Correlation With Environmental Factors

The indoor environment can significantly influence microbial diversity, particularly with respect to allergens and other indoor conditions impacting health and overall well‐being of the residents. This correlation is shaped by various factors, such as the house age, including building materials, ventilation, moisture and humidity, as well as occupancy patterns and behaviour.

Building materials significantly influence indoor microbial diversity. For instance, wood flooring supports distinct microbial populations compared with synthetic materials, while textiles and firewood enhance bacterial and fungal diversity (Coombs et al. 2018; Cox et al. 2021; Fu, Li, et al. 2020; Fu, Norbäck, et al. 2020). Ventilation also plays a critical role in shaping indoor microbial diversity, especially during winter. Inadequate ventilation and cleaning can lead to pollutant and moisture accumulation, fostering microbial growth (Mäki et al. 2021; Vidal‐Quist et al. 2021). Effective ventilation, whether natural or mechanical, facilitates air exchange between indoor and outdoor environments, increasing microbial diversity—homes with poor ventilation often have elevated indoor microbial concentrations, while those with open windows introduce outdoor microorganisms like *Cladosporium* and *Alternaria*, enhancing fungal diversity (Asif et al. 2019; Weikl et al. 2016; Hui et al. 2019; Valkonen et al. 2015, 2018). This is especially pronounced in vegetated areas (Lee, Yang, et al. 2021; Mäki et al. 2021; Tong et al. 2017).

Seasonality and moisture are also very important factors shaping indoor microbial diversity, showing significant variations throughout the year. Moisture has a crucial role in fostering fungal growth indoors‐ environments with visible dampness or water damage support higher fungal diversity, particularly species like *Aspergillus* and *Cladosporium* (Niemeier‐Walsh et al. 2021). High humidity especially facilitates the growth of allergenic and pathogenic fungal species such as *Candida*, *Rhizopus*, and *Epicoccum*, potentially increasing the risk of respiratory health issues, including asthma (Cox et al. 2021; Lee, Wyss, et al. 2021; Shabankarehfard et al. 2017). Outdoor airborne fungi such as *Aspergillus*, *Cladosporium*, and *Basidiomycetes* are more prevalent in warmer months and can enter indoor environments through ventilation systems (Gupta et al. 2020; Richardson et al. 2019; Shabankarehfard et al. 2017). Conversely, colder months stabilise indoor microbial diversity, with bacterial communities dominated by human‐associated microbes and a decrease in fungal diversity (Sitarik et al. 2018; Valkonen et al. 2018). Overall, warmer, more humid conditions promote microbial diversity, while drier, colder conditions reduce it, highlighting the dynamic influence of seasonality on indoor microbial ecosystems (Coombs et al. 2018; Estensmo et al. 2021).

Another factor that greatly influences microbial diversity in indoor environments is human occupancy and behaviour. Spaces with higher occupancy, like classrooms and homes with multiple members, are linked to increased bacterial diversity, including *Staphylococcus* and *Streptococcus* (especially in highly used areas, such as child bedrooms and living rooms). Activities such as cooking and cleaning further introduce and disperse bacteria (Weikl et al. 2016; Ciaccio et al. 2015; Eiffert et al. 2016; Ludwig et al. 2017). Bacterial diversity also increases in high‐occupancy public settings, such as cafeterias and daycare centres, with cleaning practices as a significant factor for microbial diversity and abundance (Asif et al. 2019; Coombs et al. 2018; Loo et al. 2018). It has also been shown that homes with pets, especially in farming environments, show higher bacterial diversity due to the introduction of animal‐associated microbes. Bacteria such as *Moraxella*, *Porphyromonas*, *Sutterella*, *Clostridium*, and *Mycobacterium* are more prevalent in pet‐owning households, contributing to enriched microbial communities linked to potential health benefits, including lower rates of atopic diseases (Gupta et al. 2020; Richardson et al. 2019; Sitarik et al. 2018; Loo et al. 2018; Valkonen et al. 2015). The presence of dogs significantly alters indoor dust microbiota, increasing bacterial diversity that can persist for up to a year post‐introduction. Studies show pet‐owning households have higher bacterial and fungal loads, beneficially impacting IAQ and human health (Cox et al. 2021; Lee, Yang, et al. 2021). However, pet ownership can also introduce zoonotic microorganisms that may pose health risks. Pets can harbour and transmit opportunistic pathogens such as *Salmonella*, *Campylobacter*, and *Toxocara*, potentially increasing exposure to zoonotic infections, particularly in children or immunocompromised individuals (Chomel and Sun 2011; Stull et al. 2015). Maintaining appropriate hygiene and regular veterinary care is essential to balance these microbial benefits with potential health risks.

### 3.6 Other Findings

#### 3.6.1 Indoor Air Quality and Other Air Pollutants

Air pollutants such as NO_2_, SO_2_, and PM10 have been shown to affect the diversity and composition of the indoor microbiome significantly (Isa et al. 2022; Sun et al. 2022). Fungal communities were more strongly influenced by fine particulate matter than bacterial communities (Ding et al. 2020). A previous study identified the following as the most important determinants of indoor fungal community composition: outdoor versus indoor environment (7.6%), climate (4.2%), building features (2.1%), and occupant characteristics (1.9%) (Martin‐Sanchez et al. 2021). According to other studies, temperature, room type, and humidity are key factors shaping microbial communities in indoor environments (Estensmo et al. 2021).

Temperature was found to be a strong predictor of total culturable fungi in house dust (Chew et al. 2001). In classrooms, higher indoor temperatures increased bacterial evenness, while outdoor temperatures had the opposite effect, reducing bacterial evenness but increasing bacterial load (Lee, Yang, et al. 2021).

Relative humidity and temperature were significantly and positively associated with total fungal concentration (Cochran et al. 2022). Elevation in temperature and relative humidity were also linked to a reduction in indoor microbial diversity (Park et al. 2021, 2022). Classrooms that had water damage exhibited greater bacterial richness (Park et al. 2021), though they had lower levels of protective microbes (Fu, Ou, et al. 2021a). A negative correlation was found between indoor mould amount and indoor temperature, while a positive correlation was found between indoor mould amount and indoor moisture ratio (Celtik et al. 2011). Exposure to high humidity and mould damage has also been linked to changes in certain fungal and bacterial species (Cox et al. 2021; Jayaprakash et al. 2017).

Natural ventilation had a relatively minor impact on microbial structure, with bacteria being more influenced than fungi (Núñez and García 2022; Weikl et al. 2016; Yang et al. 2022). Cleaning practices were also associated with changes in bacterial composition, showing that room cleaning methods can significantly alter indoor microbial populations (Hickman et al. 2022).

Finally, indoor bioaerosol communities have been found to be significantly affected by outdoor conditions, especially in the presence of haze and pollution (Zhou et al. 2021).

#### 3.6.2 Molecular and Mechanistic Limitations in Linking Indoor Microbiomes to Disease

A growing body of literature has reported associations between moisture‐damaged indoor environments and various adverse health symptoms, including respiratory irritation, wheezing, coughing, asthma exacerbation, and general malaise. However, identifying robust biological mechanisms linking microbial exposures to health effects remains a significant challenge.

Ndika et al. (2018) examined gene expression profiles in nasal mucosa and peripheral blood cells of teachers working in either moisture‐damaged or reference school buildings. Despite a higher prevalence of upper and lower respiratory symptoms among teachers in moisture‐damaged environments, the study found no differentially expressed genes in blood samples, and only a limited set of modestly altered genes in nasal tissue. The transcriptomic differences were not strong enough to define clear molecular pathways or biomarkers that could explain the reported symptoms. These findings highlight the difficulty in capturing subtle biological effects from low‐level or chronic environmental exposures using bulk gene expression profiling.

Suojalehto et al. (2021) conducted a detailed case–control transcriptomic study to investigate molecular mechanisms underlying adult‐onset asthma associated with exposure to damp and mouldy buildings. The study compared nasal epithelial gene expression among four groups: individuals with asthma attributed to damp buildings (AAD), those with asthma unrelated to dampness exposure (AND), patients with idiopathic environmental intolerance (IEI), and healthy controls. Transcriptomic profiling revealed that the AND group exhibited upregulation of canonical inflammatory genes—particularly those associated with Th2‐type immune responses, epithelial cytokines, and chemokines, consistent with typical allergic asthma. In contrast, the AAD and IEI groups showed only modest and heterogeneous transcriptomic changes. In the AAD group, weak activation was noted in a limited number of genes related to epithelial barrier function and immune signalling, but these changes lacked interindividual consistency. Moreover, gene expression profiles in the AAD group more closely resembled those of the IEI group, which is often considered to reflect non‐inflammatory or psychogenic mechanisms. Importantly, the study did not identify a distinct or reproducible transcriptomic signature in the AAD group, despite the presence of clear clinical symptoms. These findings suggest that asthma attributed to damp indoor environments may not be driven by overt mucosal inflammation, but rather by multifactorial and potentially indirect pathways including epithelial dysregulation, individual susceptibility, neuroimmune interactions, or co‐exposures to microbial and chemical agents.

Together, these two transcriptomic studies underscore the mismatch between subjective health complaints and objective molecular biomarkers in the context of indoor air‐related illness. They also reflect broader limitations in current research, including insufficient exposure characterisation, lack of sensitive or validated biomarkers, and the influence of psychosocial or behavioural confounders. To advance understanding, future research must incorporate longitudinal designs, high‐resolution exposure profiling, and multi‐omics strategies to unravel the complex interactions between the indoor microbiome and host responses.

## 4 Discussion

This review highlights the complexity and variability in indoor microbiome research, emphasising the need for methodological standardisation to improve data comparability and the interpretability of findings. Across over 100 studies, researchers used a wide range of sampling strategies, analytical tools, and sequencing platforms. This heterogeneity underscores a key limitation in the current field: the lack of standardised procedures from the field (e.g., contextual information retrieved and sampling methods) to the lab (e.g., lysis protocols or commercial kits) limits the reproducibility and scalability of results across different environments and populations.

A strength of this review lies in its comprehensive scope, encompassing diverse geographic regions, age groups, and indoor environments, from homes and kindergartens to offices and healthcare settings. This diversity offers a thorough understanding of how environmental, architectural, and behavioural factors impact indoor microbial communities. Traditional methods for studying indoor microbiomes, such as culture‐based techniques or targeted qPCR, have provided foundational insights but are limited in scope, sensitivity, and taxonomic resolution. Many indoor microorganisms are non‐culturable or present at low abundance, leading to underestimation of microbial diversity and incomplete ecological interpretation. Notably, the use of advanced sequencing techniques, such as 16S rRNA and ITS amplicon sequencing, especially on platforms like Illumina MiSeq, provides a robust backbone for microbial profiling. Moreover, recent advances in long‐read sequencing platforms such as PacBio and ONT further enhance resolution. However, the limited adoption of shotgun metagenomics, which reveals the functional diversity of microbial communities by sequencing all genomic DNA contained in a sample, remains a gap. Therefore, integrating both approaches, as highlighted by recent work (Yang et al. 2025; Yun et al. 2025), can be essential to move beyond describing microbial diversity to understanding how indoor microbes relate to the built environment and human health.

Ecological findings demonstrate a clear influence of environmental conditions such as ventilation, humidity, temperature, and occupant behaviour on microbial diversity. For example, buildings constructed with natural materials, featuring regular ventilation, and those with the presence of pets tend to harbour more diverse and potentially beneficial microbial populations. Conversely, high humidity, water damage, and improper chemical cleaning practices can promote the growth of pathogenic or allergenic taxa. These findings suggest that architectural design, cleaning practices, and occupant behaviour can be modified to promote healthier indoor microbiomes.

One notable limitation is the variability in DNA extraction methods, particularly for low‐biomass samples such as air or HVAC filters. Inconsistent use of lysis protocols or commercial kits (e.g., PowerSoil, FastDNA SPIN) can lead to differential microbial recovery, which, in turn, influences downstream data interpretation. Additionally, the lack of standardisation in amplicon sequencing protocols, such as differences in amplified regions and sequencing strategies, makes it difficult to compare data across multiple projects. Another challenge is the inconsistent reporting of metadata (contextual data), such as sampling site conditions or participant health status, which hinders the ability to draw causal links between microbial exposure and health outcomes, including asthma.

Importantly, the implications of this work extend beyond academic research. As indoor environments become the dominant setting for human activity, especially following the COVID‐19 pandemic, understanding the microbiome's role in indoor air quality (IAQ) becomes increasingly critical. This review suggests that promoting microbial diversity, through the selection of building materials, ventilation design, and pet ownership, may offer protective benefits against respiratory conditions. At the same time, the use of antimicrobial cleaning agents and over‐sterilisation practices, which surged during the pandemic, may disrupt beneficial microbial exposures, potentially increasing long‐term health risks.

## 5 Future Recommendations

Future research should: (i) prioritise epidemiological longitudinal studies using multiple sampling methods to understand spatio‐temporal shifts in indoor microbiota and their links to occupant health, (ii) establish standardised operating procedures (SOPs) for microbiome sequencing, (iii) define reference extraction kits for cross‐study comparability, (iv) include multi‐omics approaches, for example, integrate functional profiling approaches, such as shotgun metagenomics and transcriptomics, to move beyond taxonomy and explore microbial function.

**FIGURE 4:**
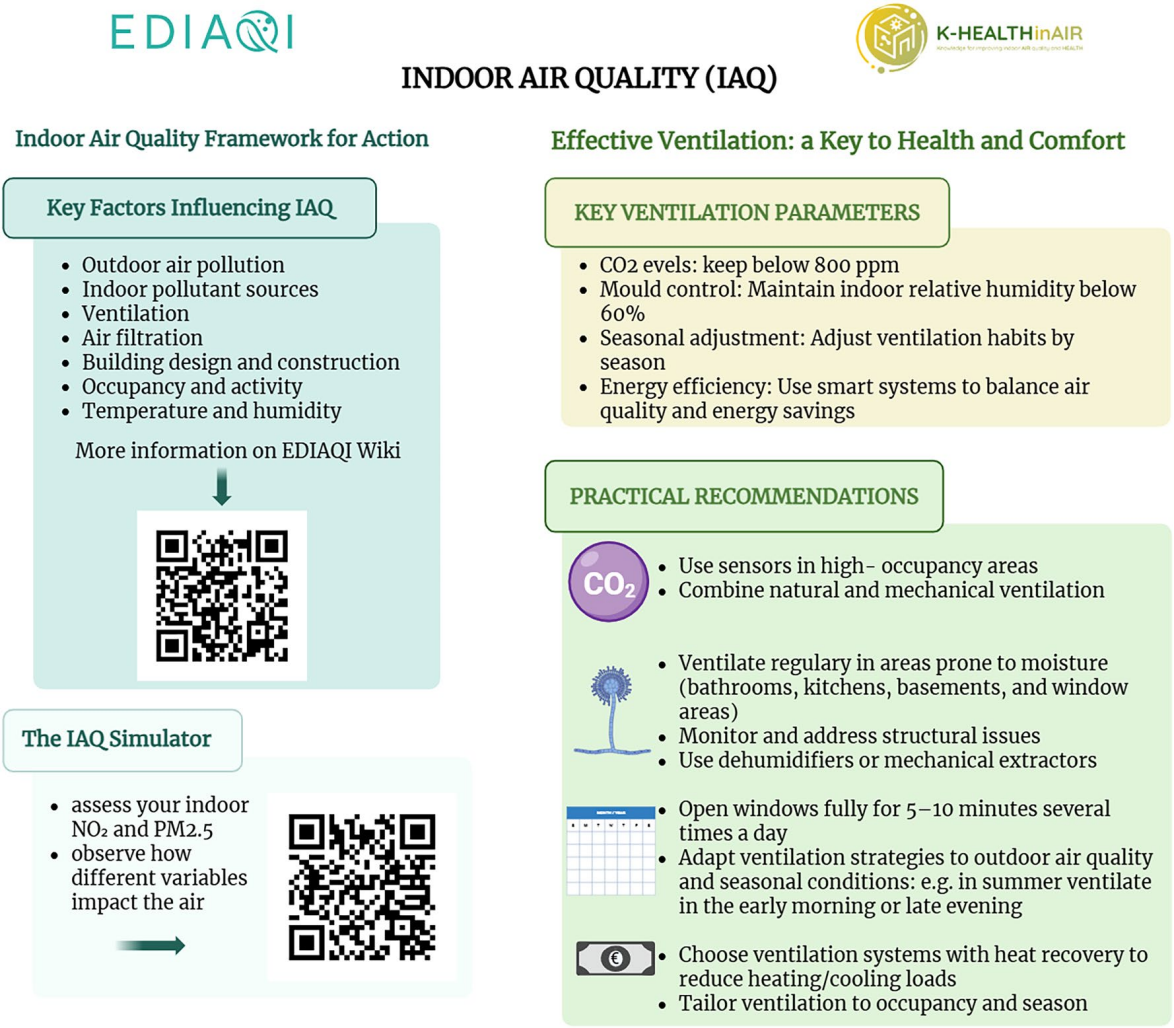
Overview of framework and ventilation guidance for improving indoor air quality.

Additionally, projects within the IDEAL cluster, addressing different aspects of indoor and outdoor air pollution, have created a range of tools designed to raise public awareness about air pollution. For example, an infographic “Effective ventilation: key to health and comfort” (Figure S1), prepared as part of the K‐HEALTHinAIR project, aims to raise public awareness of reducing the risk of respiratory illnesses by improving IAQ. This simple ventilation guide provides easy‐to‐follow instructions on how to improve indoor air exchange, considering seasonal weather conditions, as well as economic and energy aspects. With a similar aim, EDIAQI project (Lovrić et al. 2025) has created a wiki page encompassing the majority of publicly available data on IAQ and a simulation tool for air pollution risk assessment, which individuals and/or local authorities can use for preliminary risk assessment of their buildings. Figure 4 provides a visual summary.

Finally, policy makers and public health authorities should use these insights to inform IAQ regulations, building codes, and public awareness campaigns aimed at creating healthier indoor environments, especially for priority (hospitals, schools, kindergartens, etc.) and other public health important facilities that have the potential for larger gatherings. Future guidelines should emphasise smart ventilation, for example, encouraging the use of sensors and automated systems that adjust airflow in real‐time based on occupancy, pollution levels, and CO_2_ concentrations. Maintaining optimal indoor sanitary conditions, temperature and humidity between 30% and 60% is crucial to prevent mould growth and improve air quality. Guidelines for use of humidifiers and dehumidifiers, including recommendations for methods of independent assessment of device efficiency, should be consistent with local microclimate indicators, including unbiased recommendations for real‐time air quality monitoring systems use tracking a range of pollutants (PM, VOCs, CO_2_, humidity) with actionable feedback to occupants. In addition, developing sustainable building practices are key, including the use of low‐VOC and formaldehyde‐free construction and interior decoration materials, as well as promotion of green building certifications that incentivise designs focused on air quality and occupant health. Architects and engineers should also be instructed by local authorities to incorporate IAQ principles early in their design process, optimising airflow with well‐positioned windows and natural filtration systems like green walls. And, in addition to all these efforts, special attention should be put on public awareness and education of key stakeholders beside general population on improving their IAQ knowledge, together with clear communication on the health risks of indoor air pollution, particularly for vulnerable populations such as children, child‐bearing women, the older people and those with pre‐existing health conditions.

## 6 Conclusion

This review provides a comprehensive overview of current methodologies used in indoor microbiome research, highlighting significant variability in sampling approaches, matrix selection, DNA extraction protocols, and sequencing technologies. While the growing interest in characterising the indoor microbiome reflects its relevance to human health, especially respiratory conditions like asthma, the lack of standardised practices limits cross‐study comparability and reproducibility. Key insights reveal that environmental factors such as ventilation, humidity, building materials, and occupant behaviour play a crucial role in shaping microbial diversity indoors. To advance the field, future research should prioritise the development and adoption of harmonised methodological frameworks, integration of functional metagenomic analyses, and consistent metadata reporting. At a minimum, studies should clearly report sampling locations and methods (including surface type, height, and timing), use appropriate negative and positive controls, and apply consistent DNA extraction and sequencing protocols. Metadata collection should include key building characteristics (e.g., age, ventilation type, moisture, occupancy, indoor materials, cleaning product) and occupant information, where relevant. Finally, transparent data processing and sharing of raw sequences and associated metadata will facilitate cross‐study comparisons and meta‐analyses. These efforts will be essential for translating microbial ecology findings into evidence‐based public health policies and building design strategies that support healthier indoor environments.

## Author Contributions

**Iva Šunić:** conceptualization, methodology, formal analysis, investigation, data curation, writing – original draft, writing – review and editing, visualization. **Jelena Šarac:** conceptualization, investigation, formal analysis, data curation, writing – original draft, writing – review and editing. **Dubravka Havaš Auguštin:** conceptualization, formal analysis, investigation, data curation, writing – original draft, writing – review and editing. **Sofya Pozdniakova:** conceptualization, investigation, formal analysis, data curation, writing – original draft, writing – review and editing. **Robert M. W. Ferguson:** conceptualization, investigation, formal analysis, data curation, writing – original draft, writing – review and editing. **Matijana Jergović:** investigation, formal analysis, data curation, writing – original draft, writing – review and editing. **David Visentin:** formal analysis, investigation, writing – review and editing. **Sílvia Borràs:** investigation, formal analysis, data curation, writing – original draft, writing – review and editing. **Elizabeth Archer:** investigation, formal analysis, data curation, writing – original draft, writing – review and editing, visualization. **Drew K. Henderson:** investigation, formal analysis, data curation, writing – original draft, writing – review and editing, visualization. **Sandra Vitko:** investigation, formal analysis, writing – review and editing. **Adna Ašić:** investigation, formal analysis, writing – review and editing. **Anja Bošnjaković:** methodology, investigation, formal analysis, resources, data curation, writing – original draft, writing – review and editing, visualization. **Željka Maglica:** investigation, formal analysis, writing – review and editing. **Carla Viegas:** data curation, writing – original draft, writing – review and editing. **Natalija Novokmet:** investigation, formal analysis, writing – review and editing. **Nina Karlović:** methodology, investigation, formal analysis, resources, data curation, writing – original draft, writing – review and editing. **Damir Marjanović:** writing – review and editing. **Adam Muszyński:** data curation, writing – original draft, writing – review and editing. **Yuxi Liu:** data curation, writing – original draft, writing – review and editing. **Piia Karisola:** data curation, writing – original draft, writing – review and editing. **Harri Alenius:** data curation, writing – original draft, writing – review and editing. **Lukasz Krych:** writing – original draft, writing – review and editing. **Mario Lovrić:** conceptualization, methodology, writing – original draft, writing – review and editing, supervision.

## Funding

This work was supported by the European Union's Horizon Europe research and innovation programme, Grant agreement No. 101057497, Grant Agreement No. 101057779. The European Union, Grant agreement: 101056883. Swiss State Secretariat for Education, Research and Innovation, SERI grant agreement 22.00324. United Kingdom Research and Innovation, UKRI grant agreement 10040524. Australian National Health and Medical Research Council, NHMRC grant agreements APP2017786, NHMRC grant agreements APP2008813. European Union's Call on Environment and Health (HORIZON‐HLTH‐2021‐ENVHLTH‐02), 101057693.

## Conflicts of Interest

The authors declare no conflicts of interest.

## Supporting information


**Figure S1:** Effective ventilation: key to health and comfort. Infographic prepared as part of the K‐HEALTHinAIR project to raise public awareness on reducing the risk of respiratory illnesses by improving indoor air quality (IAQ).
**Table S1:** Detailed overview of the studies included in the analysis.

## Data Availability

Data sharing not applicable to this article as no datasets were generated or analysed during the current study.

## References

[emi470272-bib-0001] Adams, R. I. , H. Leppänen , A. M. Karvonen , et al. 2021. “Microbial Exposures in Moisture‐Damaged Schools and Associations With Respiratory Symptoms in Students: A Multi‐Country Environmental Exposure Study.” Indoor Air 31: 1952–1966. 10.1111/ina.12865. 34151461

[emi470272-bib-0002] Aleksic, B. , M. Draghi , S. Ritoux , et al. 2017. “Aerosolization of Mycotoxins After Growth of Toxinogenic Fungi on Wallpaper.” Applied and Environmental Microbiology 83: e01001‐17. 10.1128/AEM.01001-17. 28646113 PMC5541226

[emi470272-bib-0003] Amin, H. , T. Šantl‐Temkiv , C. Cramer , et al. 2023. “Indoor Airborne Microbiome and Endotoxin: Meteorological Events and Occupant Characteristics Are Important Determinants.” Environmental Science & Technology 57: 11750–11766. 10.1021/acs.est.3c01616. 37523308 PMC10433529

[emi470272-bib-0004] An, X.‐L. , J.‐X. Xu , M.‐R. Xu , et al. 2023. “Dynamics of Microbial Community and Potential Microbial Pollutants in Shopping Malls.” mSystems 8: e00576‐22. 10.1128/msystems.00576-22. 36602317 PMC9948725

[emi470272-bib-0005] Araujo, R. , J. P. Cabral , and A. G. Rodrigues . 2008. “Air Filtration Systems and Restrictive Access Conditions Improve Indoor Air Quality in Clinical Units: Penicillium as a General Indicator of Hospital Indoor Fungal Levels.” American Journal of Infection Control 36: 129–134. 10.1016/j.ajic.2007.02.001. 18313515

[emi470272-bib-0006] Asif, A. , M. Zeeshan , I. Hashmi , U. Zahid , and M. F. Bhatti . 2018. “Microbial Quality Assessment of Indoor Air in a Large Hospital Building During Winter and Spring Seasons.” Building and Environment 135: 68–73. 10.1016/j.buildenv.2018.03.010.

[emi470272-bib-0007] Asif, A. , M. Zeeshan , and M. Jahanzaib . 2019. “Assessment of Indoor and Outdoor Microbial Air Quality of Cafeterias of an Educational Institute.” Atmospheric Pollution Research 10: 531–536. 10.1016/j.apr.2018.09.012.

[emi470272-bib-0008] Barberán, A. , R. R. Dunn , B. J. Reich , et al. 2015. “The Ecology of Microscopic Life in Household Dust.” Proceedings of the Biological Sciences 282: 20151139. 10.1098/rspb.2015.1139. 26311665 PMC4571696

[emi470272-bib-0009] Birzele, L. T. , M. Depner , M. J. Ege , et al. 2017. “Environmental and Mucosal Microbiota and Their Role in Childhood Asthma.” Allergy 72: 109–119. 10.1111/all.13002. 27503830

[emi470272-bib-0010] Bisgaard, H. , K. Bønnelykke , and J. Stokholm . 2014. “Immune‐Mediated Diseases and Microbial Exposure in Early Life.” Clinical & Experimental Allergy 44: 475–481. 10.1111/cea.12291. 24533884

[emi470272-bib-0011] Bolyen, E. , J. R. Rideout , M. R. Dillon , et al. 2019. “Reproducible, Interactive, Scalable and Extensible Microbiome Data Science Using QIIME 2.” Nature Biotechnology 37: 852–857. 10.1038/s41587-019-0209-9. PMC7015180 31341288

[emi470272-bib-0012] Böttcher, M. F. , B. Björkstén , S. Gustafson , T. Voor , and M. C. Jenmalm . 2003. “Endotoxin Levels in Estonian and Swedish House Dust and Atopy in Infancy.” Clinical & Experimental Allergy 33: 295–300. 10.1046/j.1365-2222.2003.01562.x. 12614441

[emi470272-bib-0013] Bouillard, L. , O. Michel , M. Dramaix , and M. Devleeschouwer . 2005. “Bacterial Contamination of Indoor Air, Surfaces, and Settled Dust, and Related Dust Endotoxin Concentrations in Healthy Office Buildings.” Annals of Agricultural and Environmental Medicine 12: 187–192. 16457472

[emi470272-bib-0014] Bourzac, K. 2025. “The Probiotic Home Where Microbes Are Welcome Guests.” Nature. https://www.nature.com/immersive/d41586‐025‐03291‐2/index.html. 10.1038/d41586-025-03291-2 41087521

[emi470272-bib-0015] Cai, J. , Y. Shen , Y. Zhao , et al. 2023. “Early‐Life Exposure to PM2.5 and Sleep Disturbances in Preschoolers From 551 Cities of China.” American Journal of Respiratory and Critical Care Medicine 207: 602–612. 10.1164/rccm.202204-0740OC. 36170612

[emi470272-bib-0016] Callahan, B. J. , P. J. McMurdie , M. J. Rosen , A. W. Han , A. J. A. Johnson , and S. P. Holmes . 2016. “DADA2: High‐Resolution Sample Inference From Illumina Amplicon Data.” Nature Methods 13: 581–583. 10.1038/nmeth.3869. 27214047 PMC4927377

[emi470272-bib-0017] Celtik, C. , S. Okten , O. Okutan , et al. 2011. “Investigation of Indoor Molds and Allergic Diseases in Public Primary Schools in Edirne City of Turkey.” Asian Pacific Journal of Allergy and Immunology 29: 42–49. 21560487

[emi470272-bib-0018] Centers for Disease Control and Prevention . 2019. “2018 National Health Interview Survey (NHIS) – Current Asthma Prevalence, United States.” Centers for Disease Control and Prevention. https://www.cdc.gov/asthma/nhis/2018/table4‐1.htm.

[emi470272-bib-0019] Cervantes, R. , P. Pena , B. Riesenberger , et al. 2025. “Critical Insights on Fungal Contamination in Schools: A Comprehensive Review of Assessment Methods.” Frontiers in Public Health 13: 1557506. 10.3389/fpubh.2025.1557506. 40626168 PMC12230019

[emi470272-bib-0020] Chen, Y. , Z. Liang , G. Li , and T. An . 2024. “Indoor/Outdoor Airborne Microbiome Characteristics in Residential Areas Across Four Seasons and Its Indoor Purification.” Environment International 190: 108857. 10.1016/j.envint.2024.108857. 38954924

[emi470272-bib-0021] Chen, Y.‐P. , Y. Cui , and J.‐G. Dong . 2010. “Variation of Airborne Bacteria and Fungi at Emperor Qin's Terra‐Cotta Museum, Xi'an, China, During the “Oct. 1” Gold Week Period of 2006.” Environmental Science and Pollution Research International 17: 478–485. 10.1007/s11356-009-0161-1. 19479295

[emi470272-bib-0022] Chew, G. L. , J. Douwes , G. Doekes , et al. 2001. “Fungal Extracellular Polysaccharides, Beta (1‐>3)‐Glucans and Culturable Fungi in Repeated Sampling of House Dust.” Indoor Air 11: 171–178. 10.1034/j.1600-0668.2001.011003171.x. 11521501

[emi470272-bib-0023] Chew, G. L. , C. Rogers , H. A. Burge , M. L. Muilenberg , and D. R. Gold . 2003. “Dustborne and Airborne Fungal Propagules Represent a Different Spectrum of Fungi With Differing Relations to Home Characteristics.” Allergy 58: 13–20. 10.1034/j.1398-9995.2003.00013.x. 12580801

[emi470272-bib-0024] Chomel, B. B. , and B. Sun . 2011. “Zoonoses in the Bedroom.” Emerging Infectious Diseases 17: 167–172. 10.3201/eid1702.101070. 21291584 PMC3298380

[emi470272-bib-0025] Ciaccio, C. E. , C. Barnes , K. Kennedy , M. Chan , J. Portnoy , and L. Rosenwasser . 2015. “Home Dust Microbiota Is Disordered in Homes of Low‐Income Asthmatic Children.” Journal of Asthma 52: 873–880. 10.3109/02770903.2015.1028076. PMC4807694 26512904

[emi470272-bib-0026] Ciaccio, C. E. , K. Kennedy , C. S. Barnes , J. M. Portnoy , and L. J. Rosenwasser . 2014. “The Home Microbiome and Childhood Asthma.” Journal of Allergy and Clinical Immunology 133: AB70. 10.1016/j.jaci.2013.12.274.

[emi470272-bib-0027] Cobbold, A. T. , M. A. Crane , L. D. Knibbs , I. C. Hanigan , S. P. Greaves , and C. E. Rissel . 2022. “Perceptions of Air Quality and Concern for Health in Relation to Long‐Term Air Pollution Exposure, Bushfires, and COVID‐19 Lockdown: A Before‐And‐After Study.” Journal of Climate Change and Health 6: 100137. 10.1016/j.joclim.2022.100137. 35469247 PMC9022397

[emi470272-bib-0028] Cochran, S. J. , L. Acosta , A. Divjan , et al. 2022. “Spring Is Associated With Increased Total and Allergenic Fungal Concentrations in House Dust From a Pediatric Asthma Cohort in New York City.” Building and Environment 226: 226. 10.1016/j.buildenv.2022.109711. PMC10193533 37215628

[emi470272-bib-0029] Cole, J. R. , Q. Wang , J. A. Fish , et al. 2014. “Ribosomal Database Project: Data and Tools for High Throughput rRNA Analysis.” Nucleic Acids Research 42: D633–D642. 10.1093/nar/gkt1244. 24288368 PMC3965039

[emi470272-bib-0030] Coombs, K. , D. Taft , D. V. Ward , et al. 2018. “Variability of Indoor Fungal Microbiome of Green and Non‐Green Low‐Income Homes in Cincinnati, Ohio.” Science of the Total Environment 610–611: 212–218. 10.1016/j.scitotenv.2017.07.274. PMC6728913 28803198

[emi470272-bib-0031] Cox, J. , R. Indugula , S. Vesper , Z. Zhu , R. Jandarov , and T. Reponen . 2017. “Comparison of Indoor Air Sampling and Dust Collection Methods for Fungal Exposure Assessment Using Quantitative PCR.” Environ Sci Process Impacts 19: 1312–1319. 10.1039/c7em00257b. 28858343 PMC5884110

[emi470272-bib-0032] Cox, J. , T. Stone , P. Ryan , et al. 2021. “Associations of Observed Home Dampness and Mold With the Fungal and Bacterial Dust Microbiomes.” Environmental Science: Processes & Impacts 23: 491–500. 10.1039/D0EM00505C. 33647083

[emi470272-bib-0033] Cox, J. , T. Stone , P. Ryan , et al. 2022. “Residential Bacteria and Fungi Identified by High‐Throughput Sequencing and Childhood Respiratory Health.” Environmental Research 204: 112377. 10.1016/j.envres.2021.112377. 34800538

[emi470272-bib-0034] Dalton, K. R. , M. Lee , Z. Wang , et al. 2024. “Occupational Farm Work Activities Influence Workers' Indoor Home Microbiome.” Environmental Research 243: 117819. 10.1016/j.envres.2023.117819. 38052359 PMC10872285

[emi470272-bib-0036] Dannemiller, G. J. F. , B. P. Leaderer , and J. Peccia . 2016. “Influence of Housing Characteristics on Bacterial and Fungal Communities in Homes of Asthmatic Children.” Indoor Air 26: 179–192. 10.1111/ina.12205. 25833176 PMC4591094

[emi470272-bib-0035] Dannemiller, K. C. , J. F. Gent , B. P. Leaderer , and J. Peccia . 2016. “Indoor Microbial Communities: Influence on Asthma Severity in Atopic and Nonatopic Children.” Journal of Allergy and Clinical Immunology 138: 76–83.e1. 10.1016/j.jaci.2015.11.027. 26851966 PMC5357886

[emi470272-bib-0037] de Bont, J. , S. Jaganathan , M. Dahlquist , Å. Persson , M. Stafoggia , and P. Ljungman . 2022. “Ambient Air Pollution and Cardiovascular Diseases: An Umbrella Review of Systematic Reviews and Meta‐Analyses.” Journal of Internal Medicine 291: 779–800. 10.1111/joim.13467. 35138681 PMC9310863

[emi470272-bib-0038] Delgado‐Saborit, J. M. , V. Guercio , A. M. Gowers , G. Shaddick , N. C. Fox , and S. Love . 2021. “A Critical Review of the Epidemiological Evidence of Effects of Air Pollution on Dementia, Cognitive Function and Cognitive Decline in Adult Population.” Science of the Total Environment 757: 143734. 10.1016/j.scitotenv.2020.143734. 33340865

[emi470272-bib-0039] Dias, M. , B. Gomes , P. Pena , et al. 2024. “Filling the Knowledge Gap: Scoping Review Regarding Sampling Methods, Assays, and Further Requirements to Assess Airborne Viruses.” Science of the Total Environment 946: 174016. 10.1016/j.scitotenv.2024.174016. 38908595

[emi470272-bib-0040] Dijkhoff, I. M. , B. Drasler , B. B. Karakocak , et al. 2020. “Impact of Airborne Particulate Matter on Skin: A Systematic Review From Epidemiology to In Vitro Studies.” Particle and Fibre Toxicology 17: 35. 10.1186/s12989-020-00366-y. 32711561 PMC7382801

[emi470272-bib-0041] Ding, L.‐J. , X.‐Y. Zhou , and Y.‐G. Zhu . 2020. “Microbiome and Antibiotic Resistome in Household Dust From Beijing, China.” Environment International 139: 105702. 10.1016/j.envint.2020.105702. 32248025

[emi470272-bib-0042] Dowlatabadi, Y. , Z. E. Khajeh , M. Mohammadi , M. Sarkhosh , S. Mohammad , and M. Moezzi . 2024. “Assessment of Meteorological Factors and Air Pollution Impact on Cardiovascular Mortality Using Random Forest Analysis 2017 to 2020.” Scientific Reports 14: 31468. 10.1038/s41598-024-83185-x. 39733181 PMC11682359

[emi470272-bib-0043] Dunn, R. R. , N. Fierer , J. B. Henley , J. W. Leff , and H. L. Menninger . 2013. “Home Life: Factors Structuring the Bacterial Diversity Found Within and Between Homes.” PLoS One 8: e64133. 10.1371/journal.pone.0064133. 23717552 PMC3661444

[emi470272-bib-0044] Ege, M. J. , M. Mayer , A.‐C. Normand , et al. 2011. “Exposure to Environmental Microorganisms and Childhood Asthma.” New England Journal of Medicine 364: 701–709. 10.1056/NEJMoa1007302. 21345099

[emi470272-bib-0045] Ege, M. J. , M. Mayer , K. Schwaiger , et al. 2012. “Environmental Bacteria and Childhood Asthma.” Allergy 67: 1565–1571. 10.1111/all.12028. 22994424

[emi470272-bib-0046] Eiffert, S. , Y. Noibi , S. Vesper , et al. 2016. “A Citizen‐Science Study Documents Environmental Exposures and Asthma Prevalence in Two Communities.” Journal of Environmental and Public Health 2016: 1962901. 10.1155/2016/1962901. 28003835 PMC5143781

[emi470272-bib-0047] Estensmo, E. L. F. , L. Morgado , S. Maurice , et al. 2021. “Spatiotemporal Variation of the Indoor Mycobiome in Daycare Centers.” Microbiome 9: 220. 10.1186/s40168-021-01167-x. 34753520 PMC8576891

[emi470272-bib-0048] Estensmo, E. L. F. , S. Smebye Botnen , S. Maurice , et al. 2022. “The Indoor Mycobiomes of Daycare Centers Are Affected by Occupancy and Climate.” Applied and Environmental Microbiology 88: e0211321. 10.1128/AEM.02113-21. 35196140 PMC8939353

[emi470272-bib-0050] European Environment Agency . 2024. “Harm to Human Health From Air Pollution in Europe: Burden of Disease Status, 2024.” https://www.eea.europa.eu/en/analysis/publications/harm‐to‐human‐health‐from‐air‐pollution‐2024.

[emi470272-bib-0049] European Environment Agency . 2025. “Morbidity due to Exposure to Air Pollution (Signal).” https://www.eea.europa.eu/en/european‐zero‐pollution‐dashboards/indicators/morbidity‐due‐to‐exposure‐to‐air‐pollution‐signal‐update‐1.

[emi470272-bib-0051] EUROSTAT . 2021. “Finland: EU Country With Highest Share of Asthmatics,” Finland: EU Country with Highest Share of Asthmatics. https://ec.europa.eu/eurostat/web/products‐eurostat‐news/‐/edn‐20210924‐1.

[emi470272-bib-0052] Eze, I. C. , E. Schaffner , M. Foraster , et al. 2015. “Long‐Term Exposure to Ambient Air Pollution and Metabolic Syndrome in Adults.” PLoS One 10: e0130337. 10.1371/journal.pone.0130337. 26103580 PMC4478007

[emi470272-bib-0053] Fairs, A. , J. Agbetile , M. Bourne , et al. 2013. “Isolation of *Aspergillus fumigatus* From Sputum Is Associated With Elevated Airborne Levels in Homes of Patients With Asthma.” Indoor Air 23: 275–284. 10.1111/ina.12020. 23198683

[emi470272-bib-0054] Fairs, A. , A. J. Wardlaw , J. R. Thompson , and C. H. Pashley . 2010. “Guidelines on Ambient Intramural Airborne Fungal Spores.” Journal of Investigational Allergology & Clinical Immunology 20: 490–498. 21243933

[emi470272-bib-0055] Font‐Ribera, L. , M. Rico , M. Marí‐Dell'Olmo , et al. 2023. “Estimating Ambient Air Pollution Mortality and Disease Burden and Its Economic Cost in Barcelona.” Environmental Research 216: 114485. 10.1016/j.envres.2022.114485. 36206924

[emi470272-bib-0056] Fu, X. , Y. Li , Y. Meng , et al. 2021. “Derived Habitats of Indoor Microbes Are Associated With Asthma Symptoms in Chinese University Dormitories.” Environmental Research 194: 110501. 10.1016/j.envres.2020.110501. 33221308

[emi470272-bib-0057] Fu, X. , Y. Li , Q. Yuan , et al. 2020. “Continental‐Scale Microbiome Study Reveals Different Environmental Characteristics Determining Microbial Richness, Composition, and Quantity in Hotel Rooms.” mSystems 5: e00119‐20. 10.1128/mSystems.00119-20. 32430405 PMC7253364

[emi470272-bib-0059] Fu, X. , D. Norbäck , Q. Yuan , et al. 2020. “Indoor Microbiome, Environmental Characteristics and Asthma Among Junior High School Students in Johor Bahru, Malaysia.” Environment International 138: 105664. 10.1016/j.envint.2020.105664. 32200316

[emi470272-bib-0058] Fu, X. , D. Norbäck , Q. Yuan , et al. 2021. “Association Between Indoor Microbiome Exposure and Sick Building Syndrome (SBS) in Junior High Schools of Johor Bahru, Malaysia.” Science of the Total Environment 753: 141904. 10.1016/j.scitotenv.2020.141904. 32890872

[emi470272-bib-0060] Fu, X. , Z. Ou , and Y. Sun . 2022. “Indoor Microbiome and Allergic Diseases: From Theoretical Advances to Prevention Strategies.” Eco‐Environment & Health 1: 133–146. 10.1016/j.eehl.2022.09.002. 38075599 PMC10702906

[emi470272-bib-0061] Fu, X. , Z. Ou , M. Zhang , et al. 2021a. “Classroom Microbiome, Functional Pathways and Sick‐Building Syndrome (SBS) in Urban and Rural Schools ‐ Potential Roles of Indoor Microbial Amino Acids and Vitamin Metabolites.” Science of the Total Environment 795: 148879. 10.1016/j.scitotenv.2021.148879. 34328924

[emi470272-bib-0062] Fu, X. , Z. Ou , M. Zhang , et al. 2021b. “Indoor Bacterial, Fungal and Viral Species and Functional Genes in Urban and Rural Schools in Shanxi Province, China‐Association With Asthma, Rhinitis and Rhinoconjunctivitis in High School Students.” Microbiome 9: 138. 10.1186/s40168-021-01091-0. 34118964 PMC8199840

[emi470272-bib-0063] Fu, X. , A. Shama , D. Norbäck , et al. 2024. “Exploring the Role of Indoor Microbiome and Environmental Characteristics in Rhinitis Symptoms Among University Students.” Frontiers in Microbiomes 3: 3. 10.3389/frmbi.2024.1277177. PMC12993640 41853509

[emi470272-bib-0064] Fu, X. , Q. Yuan , X. Zhu , et al. 2021. “Associations Between the Indoor Microbiome, Environmental Characteristics and Respiratory Infections in Junior High School Students of Johor Bahru, Malaysia.” Environmental Science: Processes & Impacts 23: 1171–1181. 10.1039/d1em00115a. 34278392

[emi470272-bib-0065] Fujimura, K. E. , M. Rauch , E. Matsui , et al. 2012. “Development of a Standardized Approach for Environmental Microbiota Investigations Related to Asthma Development in Children.” Journal of Microbiological Methods 91: 231–239. 10.1016/j.mimet.2012.08.016. 22975469 PMC3615718

[emi470272-bib-0066] Furst, L. , Y. Cipoli , N. Galindo , et al. 2024. “Comprehensive Analysis of Particulate Matter, Gaseous Pollutants, and Microbiological Contamination in an International Chain Supermarket.” Environmental Pollution 363: 125236. 10.1016/j.envpol.2024.125236. 39505100

[emi470272-bib-0067] Furst, L. , Y. Cipoli , E. Yubero , et al. 2025. “Indoor Air Quality in a Home Improvement Store: Gaseous Pollutants, Bioburden and Particle‐Bound Chemical Constituents.” Building and Environment 277: 112908. 10.1016/j.buildenv.2025.112908.

[emi470272-bib-0068] GBD 2021 HAP Collaborators . 2025. “Global, Regional, and National Burden of Household Air Pollution, 1990–2021: A Systematic Analysis for the Global Burden of Disease Study 2021.” Lancet 405: 1167–1181. 10.1016/S0140-6736(24)02840-X. 40118081 PMC11971481

[emi470272-bib-0069] Gensollen, T. , S. S. Iyer , D. L. Kasper , and R. S. Blumberg . 2016. “How Colonization by Microbiota in Early Life Shapes the Immune System.” Science 352: 539–544. 10.1126/science.aad9378. 27126036 PMC5050524

[emi470272-bib-0070] Gilbert, J. A. , and E. M. Hartmann . 2024. “The Indoors Microbiome and Human Health.” Nature Reviews. Microbiology 22: 742–755. 10.1038/s41579-024-01077-3. 39030408

[emi470272-bib-0071] Gilbert, J. A. , and B. Stephens . 2018. “Microbiology of the Built Environment.” Nature Reviews. Microbiology 16: 661–670. 10.1038/s41579-018-0065-5. 30127345

[emi470272-bib-0072] Global Initiative for Asthma . “Pocket Guide for Asthma Management and Prevention.” https://GinasthmaOrg. 2015.

[emi470272-bib-0073] Guo, J. , Y. Xiong , T. Kang , Z. Xiang , and C. Qin . 2020. “Bacterial Community Analysis of Floor Dust and HEPA Filters in Air Purifiers Used in Office Rooms in ILAS, Beijing.” Scientific Reports 10: 6417. 10.1038/s41598-020-63543-1. 32286482 PMC7156680

[emi470272-bib-0074] Gupta, S. , M. H. Hjelmsø , J. Lehtimäki , et al. 2020. “Environmental Shaping of the Bacterial and Fungal Community in Infant Bed Dust and Correlations With the Airway Microbiota.” Microbiome 8: 115. 10.1186/s40168-020-00895-w. 32767985 PMC7414761

[emi470272-bib-0075] Hanson, B. , Y. Zhou , E. J. Bautista , et al. 2016. “Characterization of the Bacterial and Fungal Microbiome in Indoor Dust and Outdoor Air Samples: A Pilot Study.” Environ Sci Process Impacts 18: 713–724. 10.1039/c5em00639b. 27213188 PMC5015483

[emi470272-bib-0076] Hassan, A. , M. Zeeshan , and M. Bhatti . 2021. “Indoor and Outdoor Microbiological Air Quality in Naturally and Mechanically Ventilated University Libraries.” Atmospheric Pollution Research 12: 101136. 10.1016/j.apr.2021.101136.

[emi470272-bib-0077] Hickman, B. , P. V. Kirjavainen , M. Täubel , W. M. de Vos , A. Salonen , and K. Korpela . 2022. “Determinants of Bacterial and Fungal Microbiota in Finnish Home Dust: Impact of Environmental Biodiversity, Pets, and Occupants.” Frontiers in Microbiology 13: 1011521. 10.3389/fmicb.2022.1011521. 36419417 PMC9676251

[emi470272-bib-0078] Hoisington, A. , J. Maestre , M. King , J. Siegel , and K. Kinney . 2014. “Impact of Sampler Selection on the Characterization of the Indoor Microbiome via High‐Throughput Sequencing.” Building and Environment 80: 274–282. 10.1016/j.buildenv.2014.04.021.

[emi470272-bib-0079] Hoisington, A. J. , C. E. Stamper , K. L. Bates , et al. 2023. “Human Microbiome Transfer in the Built Environment Differs Based on Occupants, Objects, and Buildings.” Scientific Reports 13: 6446. 10.1038/s41598-023-33719-6. 37081054 PMC10116103

[emi470272-bib-0080] Hui, N. , A. Parajuli , R. Puhakka , et al. 2019. “Temporal Variation in Indoor Transfer of Dirt‐Associated Environmental Bacteria in Agricultural and Urban Areas.” Environment International 132: 105069. 10.1016/j.envint.2019.105069. 31400602

[emi470272-bib-0081] Hui, Y. , D. Sandris Nielsen , and L. Krych . 2025. “De Novo Clustering of Long‐Read Amplicons Improves Phylogenetic Insight Into Microbiome Data.” Gut Microbes 17: 2516703. 10.1080/19490976.2025.2516703. 40497323 PMC12160608

[emi470272-bib-0082] Isa, K. N. M. , J. Jalaludin , Z. Hashim , L. T. L. Than , J. H. Hashim , and D. Norbäck . 2022. “Fungi Composition in Settled Dust Associated With Fractional Exhaled Nitric Oxide in School Children With Asthma.” Science of the Total Environment 853: 158639. 10.1016/j.scitotenv.2022.158639. 36089033

[emi470272-bib-0083] Jarma, D. , J. P. Maestre , J. Sanchez , et al. 2024. “Participant‐Collected Household Dust for Assessing Microorganisms and Semi‐Volatile Organic Compounds in Urban Homes.” Science of the Total Environment 908: 168230. 10.1016/j.scitotenv.2023.168230. 37951260

[emi470272-bib-0084] Jayaprakash, B. , R. I. Adams , P. Kirjavainen , et al. 2017. “Indoor Microbiota in Severely Moisture Damaged Homes and the Impact of Interventions.” Microbiome 5: 138. 10.1186/s40168-017-0356-5. 29029638 PMC5640920

[emi470272-bib-0085] Jie, Y. , N. H. Ismail , X. jie , and Z. M. Isa . 2011. “Do Indoor Environments Influence Asthma and Asthma‐Related Symptoms Among Adults in Homes? A Review of the Literature.” Journal of the Formosan Medical Association 110: 555–563. 10.1016/j.jfma.2011.07.003. 21930065

[emi470272-bib-0086] Jo, W.‐K. , and Y.‐J. Seo . 2005. “Indoor and Outdoor Bioaerosol Levels at Recreation Facilities, Elementary Schools, and Homes.” Chemosphere 61: 1570–1579. 10.1016/j.chemosphere.2005.04.103. 15982704

[emi470272-bib-0087] Juginović, A. , M. Vuković , I. Aranza , and V. Biloš . 2021. “Health Impacts of Air Pollution Exposure From 1990 to 2019 in 43 European Countries.” Scientific Reports 11: 22516. 10.1038/s41598-021-01802-5. 34795349 PMC8602675

[emi470272-bib-0088] Karvonen, A. M. , P. V. Kirjavainen , M. Täubel , et al. 2019. “Indoor Bacterial Microbiota and Development of Asthma by 10.5 Years of Age.” Journal of Allergy and Clinical Immunology 144: 1402–1410. 10.1016/j.jaci.2019.07.035. 31415782

[emi470272-bib-0089] Kauserud, H. , P. M. Martin‐Sanchez , E. L. Estensmo , et al. 2025. “Yeasts Prefer Daycares and Molds Prefer Private Homes.” Microbial Ecology 88: 7. 10.1007/s00248-025-02505-4. 39976768 PMC11842513

[emi470272-bib-0090] Keleb, A. , E. T. Abeje , C. Daba , et al. 2025. “The Odds of Developing Asthma and Wheeze Among Children and Adolescents Exposed to Particulate Matter: A Systematic Review and Meta‐Analysis.” BMC Public Health 25: 1225. 10.1186/s12889-025-22382-3. 40165124 PMC11959839

[emi470272-bib-0091] Kettleson, E. M. , A. Adhikari , S. Vesper , K. Coombs , R. Indugula , and T. Reponen . 2015. “Key Determinants of the Fungal and Bacterial Microbiomes in Homes.” Environmental Research 138: 130–135. 10.1016/j.envres.2015.02.003. 25707017 PMC4385485

[emi470272-bib-0092] Kim, H.‐J. , J. Hwang , and J.‐H. Park . 2025. “Long‐Term Exposure to Ambient Air Pollution and Metabolic Syndrome and Its Components.” Journal of Obesity & Metabolic Syndrome 34: 91–104. 10.7570/jomes24036. 40090381 PMC12067007

[emi470272-bib-0093] Kirjavainen, P. V. , A. M. Karvonen , R. I. Adams , et al. 2019. “Farm‐Like Indoor Microbiota in Non‐Farm Homes Protects Children From Asthma Development.” Nature Medicine 25: 1089–1095. 10.1038/s41591-019-0469-4. PMC7617062 31209334

[emi470272-bib-0094] Konya, T. , B. Koster , H. Maughan , et al. 2014. “Associations Between Bacterial Communities of House Dust and Infant Gut.” Environmental Research 131: 25–30. 10.1016/j.envres.2014.02.005. 24637181

[emi470272-bib-0095] Kristono, G. A. , C. Shorter , N. Pierse , J. Crane , and R. Siebers . 2019. “Endotoxin, Cat, and House Dust Mite Allergens in Electrostatic Cloths and Bedroom Dust.” Journal of Occupational and Environmental Hygiene 16: 89–96. 10.1080/15459624.2018.1536827. 30325697

[emi470272-bib-0096] Lane, M. , E. Oyster , Y. Luo , and H. Wang . 2025. “The Effects of Air Pollution on Neurological Diseases: A Narrative Review on Causes and Mechanisms.” Toxics 13: 207. 10.3390/toxics13030207. 40137534 PMC11946816

[emi470272-bib-0097] Lee, J.‐H. , and W.‐K. Jo . 2006. “Characteristics of Indoor and Outdoor Bioaerosols at Korean High‐Rise Apartment Buildings.” Environmental Research 101: 11–17. 10.1016/j.envres.2005.08.009. 16199028

[emi470272-bib-0098] Lee, M. , A. Kaul , J. M. Ward , et al. 2024. “House Dust Metagenome and Pulmonary Function in a US Farming Population.” Microbiome 12: 129. 10.1186/s40168-024-01823-y. 39026261 PMC11256371

[emi470272-bib-0099] Lee , A. B. Wyss , M. U. Carnes , et al. 2021. “House Dust Microbiota in Relation to Adult Asthma and Atopy in a US Farming Population.” Journal of Allergy and Clinical Immunology 147: 910–920. 10.1016/j.jaci.2020.06.013. 32615170 PMC7770060

[emi470272-bib-0100] Lee, B. G. , J. I. Yang , S. W. Geum , J.‐H. Park , and M.‐K. Yeo . 2021. “Investigation of Bacterial and Fungal Communities in Indoor and Outdoor Air of Elementary School Classrooms by 16S rRNA Gene and ITS Region Sequencing.” Indoor Air 31: 1553–1562. 10.1111/ina.12825. 33780050 PMC10230515

[emi470272-bib-0101] Leppänen, H. K. , A. Nevalainen , A. Vepsäläinen , et al. 2014. “Determinants, Reproducibility, and Seasonal Variation of Ergosterol Levels in House Dust.” Indoor Air 24: 248–259. 10.1111/ina.12078. 24883434

[emi470272-bib-0102] Leppänen, H. K. , M. Täubel , B. Jayaprakash , A. Vepsäläinen , P. Pasanen , and A. Hyvärinen . 2018. “Quantitative Assessment of Microbes From Samples of Indoor Air and Dust.” Journal of Exposure Science & Environmental Epidemiology 28: 231–241. 10.1038/jes.2017.24. 28975927

[emi470272-bib-0103] Leung, A. D. , A. M. Schiltz , C. F. Hall , and A. H. Liu . 2008. “Severe Atopic Dermatitis Is Associated With a High Burden of Environmental *Staphylococcus aureus* .” Clinical and Experimental Allergy 38: 789–793. 10.1111/j.1365-2222.2008.02964.x. 18341620

[emi470272-bib-0104] Leung, M. H. Y. , X. Tong , J. C. K. Tong , and P. K. H. Lee . 2018. “Airborne Bacterial Assemblage in a Zero Carbon Building: A Case Study.” Indoor Air 28: 40–50. 10.1111/ina.12410. 28767182

[emi470272-bib-0105] Leung, M. H. Y. , D. Wilkins , E. K. T. Li , F. K. F. Kong , and P. K. H. Lee . 2014. “Indoor‐Air Microbiome in an Urban Subway Network: Diversity and Dynamics.” Applied and Environmental Microbiology 80: 6760–6770. 10.1128/AEM.02244-14. 25172855 PMC4249038

[emi470272-bib-0106] Li, H. , P.‐Q. Liu , Q.‐P. Luo , et al. 2022. “Spatiotemporal Variations of Microbial Assembly, Interaction, and Potential Risk in Urban Dust.” Environment International 170: 107577. 10.1016/j.envint.2022.107577. 36244231

[emi470272-bib-0107] Li, J. , M. Li , F. Shen , Z. Zou , M. Yao , and C. Wu . 2013. “Characterization of Biological Aerosol Exposure Risks From Automobile Air Conditioning System.” Environmental Science & Technology 47: 10660–10666. 10.1021/es402848d. 23952908

[emi470272-bib-0108] Lin, K. , and L. C. Marr . 2020. “Humidity‐Dependent Decay of Viruses, but Not Bacteria, in Aerosols and Droplets Follows Disinfection Kinetics.” Environmental Science & Technology 54: 1024–1032. 10.1021/acs.est.9b04959. 31886650

[emi470272-bib-0109] Loo, E. X. L. , L. J. M. Chew , A. B. Zulkifli , et al. 2018. “Comparison of Microbiota and Allergen Profile in House Dust From Homes of Allergic and Non‐Allergic Subjects Results From the GUSTO Study.” World Allergy Organization Journal 11: 37. 10.1186/s40413-018-0212-5. 30534340 PMC6280478

[emi470272-bib-0110] Louisias, M. , A. Ramadan , A. S. Naja , and W. Phipatanakul . 2019. “The Effects of the Environment on Asthma Disease Activity.” Immunology and Allergy Clinics of North America 39: 163–175. 10.1016/j.iac.2018.12.005. 30954168 PMC6452888

[emi470272-bib-0111] Lovrić, M. , G. Gajski , J. Fernández‐Agüera , et al. 2025. “Evidence‐Driven Indoor Air Quality Improvement: An Innovative and Interdisciplinary Approach to Improving Indoor Air Quality.” BioFactors 51, no. 1: e2126. 10.1002/biof.2126. 39350641

[emi470272-bib-0112] Ludwig, S. , I. Jimenez‐Bush , E. Brigham , et al. 2017. “Analysis of Home Dust for Staphylococcus Aureus and Staphylococcal Enterotoxin Genes Using Quantitative PCR.” Science of the Total Environment 581: 750–755. 10.1016/j.scitotenv.2017.01.003. 28063655 PMC5587345

[emi470272-bib-0113] Maestre, J. P. , W. Jennings , D. Wylie , S. D. Horner , J. Siegel , and K. A. Kinney . 2018. “Filter Forensics: Microbiota Recovery From Residential HVAC Filters.” Microbiome 6: 22. 10.1186/s40168-018-0407-6. 29382378 PMC5791358

[emi470272-bib-0114] Mäki, J. M. , P. V. Kirjavainen , M. Täubel , et al. 2021. “Associations Between Dog Keeping and Indoor Dust Microbiota.” Scientific Reports 11: 5341. 10.1038/s41598-021-84790-w. 33674692 PMC7935950

[emi470272-bib-0115] Malinverno, L. , V. Barros , F. Ghisoni , et al. 2023. “A Historical Perspective of Biomedical Explainable AI Research.” Patterns 4: 100830. 10.1016/j.patter.2023.100830. 37720333 PMC10500028

[emi470272-bib-0116] Man, W. H. , W. A. A. de Steenhuijsen Piters , and D. Bogaert . 2017. “The Microbiota of the Respiratory Tract: Gatekeeper to Respiratory Health.” Nature Reviews. Microbiology 15: 259–270. 10.1038/nrmicro.2017.14. 28316330 PMC7097736

[emi470272-bib-0117] Martikainen, M.‐V. , T. Tossavainen , M. Täubel , K. Wolczkiewicz , A. Lähde , and M. Roponen . 2021. “Toxicological and Microbiological Characterization of Cow Stable Dust.” Toxicology In Vitro 75: 105202. 10.1016/j.tiv.2021.105202. 34166725

[emi470272-bib-0118] Martins, C. , V. Teófilo , M. Clemente , et al. 2025. “Sources, Levels, and Determinants of Indoor Air Pollutants in Europe: A Systematic Review.” Science of the Total Environment 964: 178574. 10.1016/j.scitotenv.2025.178574. 39855122

[emi470272-bib-0119] Martin‐Sanchez, P. M. , E.‐L. F. Estensmo , L. N. Morgado , et al. 2021. “Analysing Indoor Mycobiomes Through a Large‐Scale Citizen Science Study in Norway.” Molecular Ecology 30: 2689–2705. 10.1111/mec.15916. 33830574

[emi470272-bib-0120] McDonald, D. , Y. Jiang , M. Balaban , et al. 2024. “Greengenes2 Unifies Microbial Data in a Single Reference Tree.” Nature Biotechnology 42: 715–718. 10.1038/s41587-023-01845-1. PMC10818020 37500913

[emi470272-bib-0121] McMurdie, P. J. , and S. Holmes . 2013. “Phyloseq: An R Package for Reproducible Interactive Analysis and Graphics of Microbiome Census Data.” PLoS One 8: e61217. 10.1371/journal.pone.0061217. 23630581 PMC3632530

[emi470272-bib-0122] Meyer, H. W. , H. Würtz , P. Suadicani , et al. 2004. “Molds in Floor Dust and Building‐Related Symptoms in Adolescent School Children.” Indoor Air 14: 65–72. 10.1046/j.1600-0668.2003.00213.x. 14756847

[emi470272-bib-0123] Mortensen, M. S. , A. D. Brejnrod , M. Roggenbuck , et al. 2016. “The Developing Hypopharyngeal Microbiota in Early Life.” Microbiome 4: 70. 10.1186/s40168-016-0215-9. 28038686 PMC5203717

[emi470272-bib-0124] Nastasi, N. , S. R. Haines , L. Xu , et al. 2020. “Morphology and Quantification of Fungal Growth in Residential Dust and Carpets.” Building and Environment 174: 174. 10.1016/j.buildenv.2020.106774. PMC8064739 33897093

[emi470272-bib-0125] Ndika, J. , H. Suojalehto , M. Täubel , et al. 2018. “Nasal Mucosa and Blood Cell Transcriptome Profiles Do Not Reflect Respiratory Symptoms Associated With Moisture Damage.” Indoor Air 28: 721–731. 10.1111/ina.12472. 29729044

[emi470272-bib-0126] Niemeier‐Walsh, C. , P. H. Ryan , J. Meller , et al. 2021. “The Mycobiomes and Bacteriomes of Sputum, Saliva, and Home Dust.” Indoor Air 31: 357–368. 10.1111/ina.12750. 32969526

[emi470272-bib-0127] Nilsson, R. H. , K.‐H. Larsson , A. F. S. Taylor , et al. 2019. “The UNITE Database for Molecular Identification of Fungi: Handling Dark Taxa and Parallel Taxonomic Classifications.” Nucleic Acids Research 47: D259–D264. 10.1093/nar/gky1022. 30371820 PMC6324048

[emi470272-bib-0128] Noris, F. , J. A. Siegel , and K. A. Kinney . 2011. “Evaluation of HVAC Filters as a Sampling Mechanism for Indoor Microbial Communities.” Atmospheric Environment 45: 338–346. 10.1016/j.atmosenv.2010.10.017.

[emi470272-bib-0129] Núñez, A. , and A. M. García . 2022. “Effect of the Passive Natural Ventilation on the Bioaerosol in a Small Room.” Building and Environment 207: 108438. 10.1016/j.buildenv.2021.108438.

[emi470272-bib-0130] Nygaard, A. B. , and C. Charnock . 2018. “Longitudinal Development of the Dust Microbiome in a Newly Opened Norwegian Kindergarten.” Microbiome 6: 159. 10.1186/s40168-018-0553-x. 30219104 PMC6138906

[emi470272-bib-0131] Nygaard, A. B. , H. S. Tunsjø , R. Meisal , and C. Charnock . 2020. “A Preliminary Study on the Potential of Nanopore MinION and Illumina MiSeq 16S rRNA Gene Sequencing to Characterize Building‐Dust Microbiomes.” Scientific Reports 10: 3209. 10.1038/s41598-020-59771-0. 32081924 PMC7035348

[emi470272-bib-0132] O'Connor, G. T. , S. V. Lynch , G. R. Bloomberg , et al. 2018. “Early‐Life Home Environment and Risk of Asthma Among Inner‐City Children.” Journal of Allergy and Clinical Immunology 141: 1468–1475. 10.1016/j.jaci.2017.06.040. 28939248 PMC6521865

[emi470272-bib-0133] Oh, J. , S. Kim , M. S. Kim , et al. 2025. “Global, Regional, and National Burden of Asthma and Atopic Dermatitis, 1990–2021, and Projections to 2050: A Systematic Analysis of the Global Burden of Disease Study 2021.” Lancet Respiratory Medicine 13: 425–446. 10.1016/S2213-2600(25)00003-7. 40147466

[emi470272-bib-0134] Onwusereaka, C. O. , J. Jalaludin , K. N. M. Isa , S. B. A. Nordin , S. Abubakar , and V. C. P. Choo . 2024. “Targeted Metagenomics Identification of Microbiome in Preschools Exposed to Air Pollutants and Their Association With Respiratory Health Symptom, Allergy and Eczema.” Air Quality, Atmosphere and Health 17: 1777–1793. 10.1007/s11869-024-01545-y.

[emi470272-bib-0135] Oswin, H. P. , A. E. Haddrell , M. Otero‐Fernandez , et al. 2022. “The Dynamics of SARS‐CoV‐2 Infectivity With Changes in Aerosol Microenvironment.” Proceedings of the National Academy of Sciences of the United States of America 119: e2200109119. 10.1073/pnas.2200109119. 35763573 PMC9271203

[emi470272-bib-0136] Park, J.‐H. , A. R. Lemons , T. L. Croston , et al. 2022. “Mycobiota and the Contribution of Yeasts in Floor Dust of 50 Elementary Schools Characterized With Sequencing Internal Transcribed Spacer Region of Ribosomal DNA.” Environmental Science & Technology 56: 11493–11503. 10.1021/acs.est.2c01703. 35901271 PMC10183301

[emi470272-bib-0137] Park, J.‐H. , A. R. Lemons , J. Roseman , B. J. Green , and J. M. Cox‐Ganser . 2021. “Bacterial Community Assemblages in Classroom Floor Dust of 50 Public Schools in a Large City: Characterization Using 16S rRNA Sequences and Associations With Environmental Factors.” Microbiome 9: 15. 10.1186/s40168-020-00954-2. 33472703 PMC7819239

[emi470272-bib-0138] Park, J.‐H. , M. Sulyok , A. R. Lemons , B. J. Green , and J. M. Cox‐Ganser . 2018. “Characterization of Fungi in Office Dust: Comparing Results of Microbial Secondary Metabolites, Fungal Internal Transcribed Spacer Region Sequencing, Viable Culture and Other Microbial Indices.” Indoor Air 28: 708–720. 10.1111/ina.12470. PMC6215746 29729045

[emi470272-bib-0139] Pillarisetti, A. , W. Ye , and S. Chowdhury . 2022. “Indoor Air Pollution and Health: Bridging Perspectives From Developing and Developed Countries.” Annual Review of Environment and Resources 47: 197–229. 10.1146/annurev-environ-012220-010602.

[emi470272-bib-0140] Quast, C. , E. Pruesse , P. Yilmaz , et al. 2013. “The SILVA Ribosomal RNA Gene Database Project: Improved Data Processing and Web‐Based Tools.” Nucleic Acids Research 41: D590–D596. 10.1093/nar/gks1219. 23193283 PMC3531112

[emi470272-bib-0141] Račić, N. , I. Terzić , N. Karlović , et al. 2025. “Volatile Organic Compounds (VOCs) and Polycyclic Aromatic Hydrocarbons (PAHs) in Indoor Environments: A Review and Analysis of Measured Concentrations in Europe.” Indoor Air 2025: 5945455. 10.1155/ina/5945455.

[emi470272-bib-0142] Reska, T. , S. Pozdniakova , S. Borràs , et al. 2024. “Air Monitoring by Nanopore Sequencing.” ISME Communications 4: ycae099. 10.1093/ismeco/ycae099. 39081363 PMC11287864

[emi470272-bib-0143] Richardson, M. , N. Gottel , J. A. Gilbert , et al. 2019. “Concurrent Measurement of Microbiome and Allergens in the Air of Bedrooms of Allergy Disease Patients in the Chicago Area.” Microbiome 7: 82. 10.1186/s40168-019-0695-5. 31159879 PMC6547563

[emi470272-bib-0144] Rittenour, W. R. , C. E. Ciaccio , C. S. Barnes , et al. 2014. “Internal Transcribed Spacer rRNA Gene Sequencing Analysis of Fungal Diversity in Kansas City Indoor Environments.” Environ Sci Process Impacts 16: 33–43. 10.1039/c3em00441d. 24258337 PMC3966654

[emi470272-bib-0145] Rocchi, S. , G. Reboux , V. Frossard , et al. 2015. “Microbiological Characterization of 3193 French Dwellings of Elfe Cohort Children.” Science of the Total Environment 505: 1026–1035. 10.1016/j.scitotenv.2014.10.086. 25461103

[emi470272-bib-0146] Rognes, T. , T. Flouri , B. Nichols , C. Quince , and F. Mahé . 2016. “VSEARCH: A Versatile Open Source Tool for Metagenomics.” PeerJ 4: e2584. 10.7717/peerj.2584. 27781170 PMC5075697

[emi470272-bib-0147] Rook, G. 2023. “The Old Friends Hypothesis: Evolution, Immunoregulation and Essential Microbial Inputs.” Frontiers in Allergy 4: 1220481. 10.3389/falgy.2023.1220481. 37772259 PMC10524266

[emi470272-bib-0148] Ross, M. A. , L. Curtis , P. A. Scheff , et al. 2000. “Association of Asthma Symptoms and Severity With Indoor Bioaerosols.” Allergy 55: 705–711. 10.1034/j.1398-9995.2000.00551.x. 10955695

[emi470272-bib-0149] Šarac, J. , D. Havaš Auguštin , I. Šunić , et al. 2025. “Linking the Bed Dust Microbiome With Environmental Factors and Child Respiratory Health.” Annals of Human Biology 52: 2509606. 10.1080/03014460.2025.2509606. 40525799 PMC12312746

[emi470272-bib-0150] Sautour, M. , N. Sixt , F. Dalle , et al. 2009. “Profiles and Seasonal Distribution of Airborne Fungi in Indoor and Outdoor Environments at a French Hospital.” Science of the Total Environment 407: 3766–3771. 10.1016/j.scitotenv.2009.02.024. 19286244

[emi470272-bib-0151] Schloss, P. D. , S. L. Westcott , T. Ryabin , et al. 2009. “Introducing Mothur: Open‐Source, Platform‐Independent, Community‐Supported Software for Describing and Comparing Microbial Communities.” Applied and Environmental Microbiology 75: 7537–7541. 10.1128/AEM.01541-09. 19801464 PMC2786419

[emi470272-bib-0152] Sequeira, S. O. , E. Pasnak , C. Viegas , et al. 2024. “Microbial Assessment in A Rare Norwegian Book Collection: A One Health Approach to Cultural Heritage.” Microorganisms 12: 1215. 10.3390/microorganisms12061215. 38930597 PMC11206040

[emi470272-bib-0153] Sereika, M. , A. J. Mussig , C. Jiang , et al. 2025. “Genome‐Resolved Long‐Read Sequencing Expands Known Microbial Diversity Across Terrestrial Habitats.” Nature Microbiology 10: 2018–2030. 10.1038/s41564-025-02062-z. PMC12313526 40707831

[emi470272-bib-0154] Shabankarehfard, E. , A. Ostovar , S. Farrokhi , et al. 2017. “Air‐ and Dust‐Borne Fungi in Indoor and Outdoor Home of Allergic Patients in a Dust‐Storm‐Affected Area.” Immunological Investigations 46: 577–589. 10.1080/08820139.2017.1322102. 28742415

[emi470272-bib-0155] Shan, Y. , J. Guo , W. Fan , et al. 2020. “Modern Urbanization Has Reshaped the Bacterial Microbiome Profiles of House Dust in Domestic Environments.” World Allergy Organization Journal 13: 100452. 10.1016/j.waojou.2020.100452. 32884612 PMC7451671

[emi470272-bib-0156] Shan, Y. , W. Wu , W. Fan , T. Haahtela , and G. Zhang . 2019. “House Dust Microbiome and Human Health Risks.” International Microbiology 22: 297–304. 10.1007/s10123-019-00057-5. 30811000

[emi470272-bib-0157] Sitarik, A. , S. Havstad , A. Levin , et al. 2018. “Dog Introduction Alters the Home Dust Microbiota.” Indoor Air 28: 539–547. 10.1111/ina.12456. 29468742 PMC6003855

[emi470272-bib-0158] Soares, J. , D. Plass , S. Kienzler , A. González Ortiz , A. Gsella , and J. Horálek . 2024. “ETC HE Report 2024/6: Assessing the Environmental Burden of Disease Related to Air Pollution in Europe in 2022.”

[emi470272-bib-0159] Soto‐Serrano, A. , W. Li , F. M. Panah , et al. 2024. “Matching Excellence: Oxford Nanopore Technologies' Rise to Parity With Pacific Biosciences in Genome Reconstruction of Non‐Model Bacterium With High G+C Content.” Microbial Genomics 10: 001316. 10.1099/mgen.0.001316. 39526732 PMC11649196

[emi470272-bib-0160] Stocka, N. , A. Butarewicz , M. Stocki , P. Borowik , and T. Oszako . 2024. “Biological Pollution of Indoor Air, Its Assessment and Control Methods.” Encyclopedia 4: 1217–1235. 10.3390/encyclopedia4030079.

[emi470272-bib-0161] Stull, J. W. , J. Brophy , and J. S. Weese . 2015. “Reducing the Risk of Pet‐Associated Zoonotic Infections.” CMAJ 187: 736–743. 10.1503/cmaj.141020. 25897046 PMC4500695

[emi470272-bib-0162] Sun, Y. , Y. Meng , Z. Ou , et al. 2022. “Indoor Microbiome, Air Pollutants and Asthma, Rhinitis and Eczema in Preschool Children ‐ A Repeated Cross‐Sectional Study.” Environment International 161: 107137. 10.1016/j.envint.2022.107137. 35168186

[emi470272-bib-0163] Šunić, I. , D. Havaš Auguštin , J. Šarac , et al. 2025. “Associations Between Indoor Fungal Community Structures and Environmental Factors: Insights From the Evidence‐Driven Indoor Air‐Quality Improvement Study.” Journal of Fungi 11: 261. 10.3390/jof11040261. 40278082 PMC12028660

[emi470272-bib-0164] Suojalehto, H. , J. Ndika , I. Lindström , et al. 2021. “Transcriptomic Profiling of Adult‐Onset Asthma Related to Damp and Moldy Buildings and Idiopathic Environmental Intolerance.” International Journal of Molecular Sciences 22: 10679. 10.3390/ijms221910679. 34639020 PMC8508786

[emi470272-bib-0165] Tang, H. , S. Du , Z. Niu , et al. 2024. “Nasal, Dermal, Oral and Indoor Dust Microbe and Their Interrelationship in Children With Allergic Rhinitis.” BMC Microbiology 24: 505. 10.1186/s12866-024-03668-9. 39614169 PMC11606197

[emi470272-bib-0166] Täubel, M. , H. Rintala , M. Pitkäranta , et al. 2009. “The Occupant as a Source of House Dust Bacteria.” Journal of Allergy and Clinical Immunology 124: 834–840.e47. 10.1016/j.jaci.2009.07.045. 19767077

[emi470272-bib-0167] Tischer, C. , F. Weikl , A. J. Probst , M. Standl , J. Heinrich , and K. Pritsch . 2016. “Urban Dust Microbiome: Impact on Later Atopy and Wheezing.” Environmental Health Perspectives 124: 1919–1923. 10.1289/EHP158. 27232328 PMC5132631

[emi470272-bib-0168] Tong, X. , M. H. Y. Leung , D. Wilkins , and P. K. H. Lee . 2017. “City‐Scale Distribution and Dispersal Routes of Mycobiome in Residences.” Microbiome 5: 131. 10.1186/s40168-017-0346-7. 28978345 PMC5628474

[emi470272-bib-0169] Topalušić, I. , A. Stipić Marković , M. Artuković , S. Dodig , L. Bucić , and L. Lugović Mihić . 2022. “Divergent Trends in the Prevalence of Children's Asthma, Rhinitis and Atopic Dermatitis and Environmental Influences in the Urban Setting of Zagreb, Croatia.” Children 9: 1788. 10.3390/children9121788. 36553232 PMC9777289

[emi470272-bib-0170] Tran, V. V. , D. Park , and Y.‐C. Lee . 2020. “Indoor Air Pollution, Related Human Diseases, and Recent Trends in the Control and Improvement of Indoor Air Quality.” International Journal of Environmental Research and Public Health 17: 2927. 10.3390/ijerph17082927. 32340311 PMC7215772

[emi470272-bib-0171] Valkonen, M. , M. Täubel , J. Pekkanen , et al. 2018. “Microbial Characteristics in Homes of Asthmatic and Non‐Asthmatic Adults in the ECRHS Cohort.” Indoor Air 28: 16–27. 10.1111/ina.12427. 28960492

[emi470272-bib-0172] Valkonen, M. , I. M. Wouters , M. Täubel , et al. 2015. “Bacterial Exposures and Associations With Atopy and Asthma in Children.” PLoS One 10: e0131594. 10.1371/journal.pone.0131594. 26121165 PMC4488145

[emi470272-bib-0173] Vandenborght, L.‐E. , R. Enaud , C. Urien , et al. 2021. “Type 2‐High Asthma Is Associated With a Specific Indoor Mycobiome and Microbiome.” Journal of Allergy and Clinical Immunology 147: 1296–1305.e6. 10.1016/j.jaci.2020.08.035. 32926879 PMC7486598

[emi470272-bib-0174] Veillette, M. , L. D. Knibbs , A. Pelletier , et al. 2013. “Microbial Contents of Vacuum Cleaner Bag Dust and Emitted Bioaerosols and Their Implications for Human Exposure Indoors.” Applied and Environmental Microbiology 79: 6331–6336. 10.1128/AEM.01583-13. 23934489 PMC3811220

[emi470272-bib-0175] Vestergaard, D. V. , G. J. Holst , I. Basinas , et al. 2018. “Pig Farmers' Homes Harbor More Diverse Airborne Bacterial Communities Than Pig Stables or Suburban Homes.” Frontiers in Microbiology 9: 870. 10.3389/fmicb.2018.00870. 29765370 PMC5938556

[emi470272-bib-0176] Vidal‐Quist, J. C. , C. Vidal , F. Escolar , B. N. Lambrecht , S. Rombauts , and P. Hernández‐Crespo . 2021. “RNA Viruses in the House Dust Mite Dermatophagoides Pteronyssinus, Detection in Environmental Samples and in Commercial Allergen Extracts Used for In Vivo Diagnosis.” Allergy 76: 3743–3754. 10.1111/all.14884. 33914957

[emi470272-bib-0177] Viegas, C. , B. Almeida , A. Monteiro , et al. 2021. “Settled Dust Assessment in Clinical Environment: Useful for the Evaluation of a Wider Bioburden Spectrum.” International Journal of Environmental Health Research 31: 160–178. 10.1080/09603123.2019.1634799. 31240954

[emi470272-bib-0178] Viegas, C. , M. Dias , and S. Viegas . 2022. “Electrostatic Dust Cloth: A Useful Passive Sampling Method When Assessing Exposure to Fungi Demonstrated in Studies Developed in Portugal (2018–2021).” Pathogens 11: 345. 10.3390/pathogens11030345. 35335669 PMC8955157

[emi470272-bib-0179] Viegas, C. , C. Peixoto , B. Gomes , et al. 2024. “Assessment of Portuguese Fitness Centers: Bridging the Knowledge Gap on Harmful Microbial Contamination With Focus on Fungi.” Environmental Pollution 350: 123976. 10.1016/j.envpol.2024.123976. 38657893

[emi470272-bib-0180] Viegas, C. , S. Viegas , A. Gomes , M. Täubel , and R. Sabino . 2017. Exposure to Microbiological Agents in Indoor and Occupational Environments. Springer International Publishing. 10.1007/978-3-319-61688-9.

[emi470272-bib-0181] Viegas, S. , R. Assunção , C. Nunes , et al. 2018. “Exposure Assessment to Mycotoxins in a Portuguese Fresh Bread Dough Company by Using a Multi‐Biomarker Approach.” Toxins (Basel) 10: 342. 10.3390/toxins10090342. 30142887 PMC6162618

[emi470272-bib-0182] Vilcins, D. , R. C. Christofferson , J.‐H. Yoon , et al. 2024. “Updates in Air Pollution: Current Research and Future Challenges.” Annals of Global Health 90, no. 1: 9. 10.5334/aogh.4363. 38312715 PMC10836163

[emi470272-bib-0183] Wang, Z. , K. R. Dalton , M. Lee , et al. 2023. “Metagenomics Reveals Novel Microbial Signatures of Farm Exposures in House Dust.” Frontiers in Microbiology 14: 1202194. 10.3389/fmicb.2023.1202194. 37415812 PMC10321240

[emi470272-bib-0184] Weikl, F. , C. Tischer , A. J. Probst , et al. 2016. “Fungal and Bacterial Communities in Indoor Dust Follow Different Environmental Determinants.” PLoS One 11: e0154131. 10.1371/journal.pone.0154131. 27100967 PMC4839684

[emi470272-bib-0185] Whitby, C. , R. M. W. Ferguson , I. Colbeck , et al. 2022. “Chapter Three ‐ Compendium of Analytical Methods for Sampling, Characterization and Quantification of Bioaerosols.” In Advances in Ecological Research, edited by D. A. Bohan and A. Dumbrell , vol. 67, 101–229. Academic Press. 10.1016/bs.aecr.2022.09.004.

[emi470272-bib-0186] WHO . 2009. WHO Guidelines for Indoor Air Quality: Dampness and Mould. World Health Organization. 23785740

[emi470272-bib-0187] WHO . 2024. “Air Pollution 2024.” https://www.who.int/health‐topics/air‐pollution.

[emi470272-bib-0188] Yamamoto, N. , D. G. Shendell , and J. Peccia . 2011. “Assessing Allergenic Fungi in House Dust by Floor Wipe Sampling and Quantitative PCR.” Indoor Air 21: 521–530. 10.1111/j.1600-0668.2011.00732.x. 21767317 PMC7201893

[emi470272-bib-0189] Yang, J. , J. S. Kim , H. W. Jeon , J. Lee , and J. H. Seo . 2025. “Integrated Culture‐Based and Metagenomic Profiling of Airborne and Surface‐Deposited Bacterial Communities in Residential Environments.” Environmental Pollution 382: 126703. 10.1016/j.envpol.2025.126703. 40543204

[emi470272-bib-0190] Yang, J. , J.‐H. Seo , Y.‐K. Jee , Y.‐K. Kim , and J.‐R. Sohn . 2023. “Composition Analysis of Airborne Microbiota in Outdoor and Indoor Based on Dust Separated by Micro‐Sized and Nano‐Sized.” Aerosol and Air Quality Research 23: 210231. 10.4209/aaqr.210231.

[emi470272-bib-0191] Yang, J. I. L. , B. G. Lee , J.‐H. Park , and M.‐K. Yeo . 2022. “Airborne Fungal and Bacterial Microbiome in Classrooms of Elementary Schools During the COVID‐19 Pandemic Period: Effects of School Disinfection and Other Environmental Factors.” Indoor Air 32: e13107. 10.1111/ina.13107. 36168218 PMC9538906

[emi470272-bib-0192] Yun, H. , J. H. Seo , Y.‐K. Kim , and J. Yang . 2025. “Examining the Bacterial Diversity Including Extracellular Vesicles in Air and Soil: Implications for Human Health.” PLoS One 20: e0320916. 10.1371/journal.pone.0320916. 40168325 PMC11960916

[emi470272-bib-0193] Zhao, J. , T. He , F. Wang , and W. Liu . 2024. “Association of Prenatal and Postnatal Exposure to Air Pollution With Clinically Diagnosed Attention Deficit Hyperactivity Disorder: A Systematic Review.” Frontiers in Public Health 12: 1396251. 10.3389/fpubh.2024.1396251. 38855453 PMC11157082

[emi470272-bib-0194] Zhao, Y. , L. Li , W. Zhang , et al. 2025. “Associations of Indoor Airborne Microbiome With Systemic Inflammation in the Context of Indoor Particulate Matter Pollution and the Metabolic Mechanisms.” Journal of Environmental Sciences 159: 187–198. 10.1016/j.jes.2025.04.022. 41005875

[emi470272-bib-0195] Zhou, F. , M. Niu , Y. Zheng , et al. 2021. “Impact of Outdoor Air on Indoor Airborne Microbiome Under Hazy Air Pollution: A Case Study in Winter Beijing.” Journal of Aerosol Science 156: 105798. 10.1016/j.jaerosci.2021.105798.

