# Supplementary material for: The Indoor Microbiome: Sampling, Analysis and Emerging Trends
Source: Environ Microbiol Rep. 2026 Apr 7;18(2):e70272. doi: 10.1111/1758-2229.70272 (PMC13054241; doi:10.1111/1758-2229.70272)
Supplement: Supplementary file 1 — Figure S1: Effective ventilation: key to health and comfort. Infographic prepared as part of the K‐HEALTHinAIR project to raise public awareness on reducing the risk of respiratory illnesses by improving indoor air quality (IAQ). Table S1: Detailed overview of the studies included in the analysis. [file EMI4-18-e70272-s001.docx]

# 8. Supplement

Supplement Table 1. Detailed overview of the studies included in the analysis.

| Author | Year | Study design | Metabarcoding target | Targeted microorganisms | Country | Citations |
| --- | --- | --- | --- | --- | --- | --- |
| Amin et al. | 2023 | Cross-sectional study evaluating environmental determinants affecting bacterial abundance, diversity, and endotoxin levels related to asthma and allergies. | 16S | bacteria | Denmark, Norway, Iceland, Estonia, and Sweden. | 10.1021/acs.est.3c01616 |
| An et al. | 2023 | Cross-sectional study of dynamics of microbial communities and potential pollutants in shopping malls. | 16S and ITS | bacteria, fungi | China | 10.1128/msystems.00576-22 |
| Araujo et al. | 2008 | Prospective comparative study of fungal diversity and composition in relation to air filters and access conditions in a hospital. | Not performed | fungi | Portugal | 10.1016/j.ajic.2007.02.001 |
| Asif at al. | 2019 | Cross-sectional study monitoring microbial air quality in educational institute cafeterias. | Not performed | bacteria, fungi | Pakistan | 10.1016/j.apr.2018.09.012 |
| Asif et al. | 2018 | Prospective comparative study evaluating seasonal variation in airborne bacteria and fungi in different locations within a Pakistani hospital. | 16S (most frequently observed colonies) | bacteria, fungi | Pakistan | 10.1016/j.buildenv.2018.03.010 |
| Barberán et al. | 2015 | Cross-sectional study collecting dust samples from inside and outside homes to compare indoor and outdoor microbial communities. | 16S (V4) and ITS1 | bacteria, fungi | USA | 10.1098/rspb.2015.1139 |
| Birzele et al. | 2017 | Cross-sectional study on genetic and environmental causes of asthma in European rural children aged 6–12. | 16S (V3–V5) | bacteria | Austria, Germani, Switzerland | 10.1111/all.13002 |
| Böttcher et al. | 2003 | Prospective cohort study measuring endotoxin concentrations in dust samples from children's mattresses and carpets in Estonia and Sweden. | Not performed | na | Estonia and Sweden | 10.1046/j.1365-2222.2003.01562.x |
| Celtik et al. | 2011 | Cross-sectional study measuring indoor mold concentrations and their relation to allergic/respiratory symptoms in school children. | Not performed | fungi | Turkey | Not available |
| Chen et al. | 2010 | Cross-sectional study of airborne bacteria and fungi at Emperor Qin's Terra-Cotta Museum during Golden Week 2006. | Not performed | bacteria, fungi | China | 10.1007/s11356-009-0161-1 |
| Chen et al. | 2024 | Seasonal study of indoor/outdoor airborne microbiomes in residential homes. | 16S and ITS | bacteria, fungi | China | 10.1016/j.envint.2024.108857 |
| Chew et al. | 2001 | Longitudinal observational study of associations between EPS, b (1»3)-glucans, and culturable fungi in Dutch home dust. | Not performed | fungi | The Netherlands | 10.1034/j.1600-0668.2001.011003171.x |
| Chew et al. | 2003 | Cross-sectional study associating dustborne and airborne fungi in bedrooms of homes in Boston, USA. | Not performed | fungi | USA | 10.1034/j.1398-9995.2003.00013.x |
| Ciaccio et al. | 2014 | Cross-sectional study characterizing bacterial communities in house dust collected from homes of asthmatic and non-asthmatic children using PhyloChip microarray technology. | 16S rRNA | bacteria | USA | 10.1016/j.jaci.2013.12.274 |
| Ciaccio et al. | 2015 | Cross-sectional study characterizing indoor bacterial/fungal communities and their associations with the home environment in asthmatic children's homes. | Not performed | bacteria | USA | 10.3109/02770903.2015.1028076. |
| Cochran et al. | 2022 | Cross-sectional study associating indoor total and allergenic fungal species in dust with seasonal changes. | 18S rRNA and ITS1 | fungi | USA | 10.1016/j.buildenv.2022.109711 |
| Coombs et al. | 2018 | Prospective comparative study observing the impact of "Green" housing on the indoor mycobiome in low-income homes. | ITS1 | fungi | USA | 10.1016/j.scitotenv.2017.07.274. |
| Cox et al. | 2017 | Cross-sectional study comparing sampling methods by observing fungal species concentrations using the Environmental Relative Moldiness Index. | Not performed | fungi | USA | 10.1039/c7em00257b. |
| Cox et al. | 2021 | Cross-sectional study evaluating the effects of dampness and mold on fungal and bacterial microbiomes. | ITS1 and 16S (V4) | bacteria, fungi | USA | 10.1039/d0em00505c |
| Cox et al. | 2022 | Longitudinal observational study examining and comparing the environmental microbiota from dust and children's respiratory health outcomes. | 16S and ITS | bacteria, fungi | USA | 10.1016/j.envres.2021.112377 |
| Dalton et al. | 2024 | Case-control study using metagenomic sequencing of vacuumed bedroom dust in a farming cohort. | whole genome | bacteria | USA | 10.1016/j.envres.2023.117819 |
| Dannemiller et al. | 2016a | Cross-sectional study looking at associations of indoor bacterial/fungal communities with the home environment and its occupants. | 16S (V4) and ITS | bacteria, fungi | USA | 10.1111/ina.12205 |
| Dannemiller et al. | 2016b | Cross-sectional study examining associations between household microbial exposures and childhood asthma severity by atopic status. | 16S and ITS | bacteria, fungi | USA | 10.1016/j.jaci.2015.11.027. |
| Ding et al. | 2020 | Cross-sectional study of geographical distribution patterns of microbiomes and antibiotic resistomes in household environments. | 16S and ITS | bacteria, fungi | China | 10.1016/j.envint.2020.105702 |
| Ege et al. | 2011 | Cross-sectional study associating microbial exposure with protective effects against childhood asthma and atopy in the PARSIFAL study. | Not performed | bacteria | Germany, Austria, Switzerland | 10.1056/NEJMoa1007302. |
| Ege et al. | 2012 | Cross-sectional study associating indoor bacteria with protective effects on childhood asthma in farm children. | Not performed | bacteria | Germany | 10.1111/all.12028. |
| Eiffert et al. | 2016 | Community-based participatory research study on asthma, incorporating epidemiological cross-sectional surveys and ecological data. | Not performed | fungi | USA | 10.1155/2016/1962901 |
| Estensmo et al. | 2021 | Longitudinal observational study of spatiotemporal variation in the indoor mycobiome in daycare centers. | ITS | fungi | Norway | 10.1186/s40168-021-01167-x |
| Estensmo et al. | 2022 | Cross-sectional study comparing outdoor and indoor mycobiomes in daycare centers across different climates. | ITS | fungi | Norway | 10.1128/aem.02113-21 |
| Fairs et al. | 2013 | Cross-sectional study exploring the relationship between indoor fungal levels and respiratory outcomes in patients with asthma. | Not performed | fungi | UK | 10.1111/ina.12020 |
| Fu et al. | 2024 | Cross-sectional study of bacterial diversity in university dormitory rooms. | 16S | bacteria | China | 10.3389/frmbi.2024.1277177 |
| Fu et al. | 2020a | Cross-sectional study of hotel rooms- bacterial and fungal compositions. | Not performed | bacteria | 19 countries in Asia (Malaysia, Thailand, United Arab Emirates, India, China, Taiwan, Japan, South Korea), and Europe (Italy, Poland, Switzerland, UnitedKingdom, Netherlands, Norway, France, Austria, Belgium, Spain, Denmark) | 10.1128/msystems.00119-20 |
| Fu et al. | 2020b | Cross-sectional study collecting and analysing floor dust from junior high schools for bacterial and fungal composition studies. | 16S and ITS | bacteria, fungi | Malaysia | 10.1016/j.envint.2020.105664 |
| Fu et al. | 2021a | Cross-sectional study associating between indoor microbiome and respiratory infections- occurence of respiratory infections in classrooms and dust samples collection. | Not performed | bacteria, fungi | Malaysia | 10.1016/j.scitotenv.2020.141904 |
| Fu et al. | 2021b | Cross-sectional study investigating associations between the indoor microbiome, environmental characteristics, and respiratory infections. | 16S and ITS | bacteria, fungi | Malaysia | 10.1039/d1em00115a |
| Fu et al. | 2021c | Cross-sectional study involving questionnaire surveys and shotgun metagenomic sequencing of indoor dust. | Not performed | bacteria, fungi, viruses | China | 10.1016/j.scitotenv.2021.148879 |
| Fu et al. | 2021d | Cross-sectional study collecting dust from dormitory rooms and surveying students for asthma symptoms. | Not performed | bacteria | China | 10.1016/j.envres.2020.110501 |
| Fu et al. | 2021e | Cross-sectional study characterizing the indoor microbiome using metagenomics for functional profiling and potential health effects. | Not performed | bacteria, fungi, viruses | China | 10.1186/s40168-021-01091-0 |
| Fujimura et al. | 2012 | Pilot study for development of guidelines for high-resolution microbiome profiling of house dust samples in asthma-risk cohorts. | 16S | bacteria | USA | 10.1016/j.mimet.2012.08.016 |
| Guo et al. | 2020 | Cross-sectional study investigating bacterial communities in HEPA filters and floor dust within office rooms. | 16S (V3–V4) and ITS1 | bacteria | China | 10.1038/s41598-020-63543-1 |
| Gupta et. al. | 2020 | Prospective cohort study of indoor bed dust microbiota and early airway microbiome colonization in infants. | ITS2 and 16S (V3–V4) | bacteria, fungi | Denmark | 10.1186/s40168-020-00895-w |
| Hanson et al. | 2016 | Pilot study characterizing bacterial and fungal microbiomes in environmental samples related to human health. | 16S (V1–V3) and ITS | bacteria, fungi | USA (Boston) | 10.1039/c5em00639b. |
| Hassan et al. | 2021 | Cross-sectional study assessing indoor and outdoor microbial air quality in university libraries and their relation to ventilation systems. | 16S and ITS | bacteria, fungi | Pakistan | 10.1016/j.apr.2021.101136 |
| Hickman et al. | 2022 | Cross-sectional study characterizing indoor microbiota by amplicon sequencing, highlighting the influence of outdoor and inhabitant-derived bacteria. | 16S and ITS | bacteria, fungi | Finland | 10.3389/fmicb.2022.1011521 |
| Hoisington et al. | 2014 | Cross-sectional study assessing impact of sampler selection on indoor microbiome characterization. | 16S and ITS | bacteria, fungi | USA | 10.1016/j.buildenv.2014.04.021 |
| Hui et al. | 2019 | Longitudinal observational study examining how season, garden diversity, and animal ownership influence the indoor transfer of environmental bacteria via doormats in rural and urban households. | 16S | bacteria | Finland | 10.1016/j.envint.2019.105069 |
| Isa et al. | 2022 | Cross-sectional study evaluating associations between fungal composition in settled dust and fractional exhaled nitric oxide (FeNO) levels in asthmatic children. | ITS | fungi | Malaysia | 10.1016/j.scitotenv.2022.158639 |
| Jarma et al. | 2024 | Cross-sectional study comparing participant-collected and researcher-collected HVAC dust samples. | 16S and ITS | bacteria, fungi | USA | 10.1016/j.scitotenv.2023.168230 |
| Jayaprakash et al. | 2017 | Prospective intervention study that collected and analysed floor dust and airborne settled dust from moisture-damaged homes before and after interventions. | ITS1-2 and 16S (V4) | bacteria, fungi | Finland | 10.1186/s40168-017-0356-5 |
| Jo & Seo | 2005 | Cross-sectional study measuring bacterial and fungal concentrations in indoor and outdoor air from various microenvironments. | Not performed | bacteria, fungi | Republic of Korea | 10.1016/j.chemosphere.2005.04.103 |
| Karvonen et al. | 2019 | Prospective cohort study assessing whether indoor bacterial genera predict asthma development. | 16S (V4) | bacteria, fungi | Finland | 10.1016/j.jaci.2019.07.035 |
| Kauserud et al. | 2025 | Comparative study of indoor mycobiomes in children’s daycare centers and private homes. | ITS2 | fungi | Norway | 10.1007/s00248-025-02505-4 |
| Kettleson et al. | 2015 | Cross-sectional study analysing household factors impacting the microbiome of homes. | 16S and ITS | bacteria, fungi | USA | 10.1016/j.envres.2015.02.003 |
| Kirjavainen et al. | 2019 | Prospective cohort study of farm-like indoor microbiota in non-farm homes and its protective effects on children from asthma. | Not performed | bacteria, fungi | Finland and Germany | 10.1038/s41591-019-0469-4. |
| Konya et al. | 2014 | Descriptive study assessing associations between bacteria in house dust and nascent fecal microbiota in infants. | 16S (V5-7) | bacteria | Canada | 10.1016/j.envres.2014.02.005 |
| Lee & Jo | 2006 | Cross-sectional study measuring bacterial and fungal concentrations in indoor and outdoor air from residential apartments. | Not performed | bacteria, fungi | Republic of Korea | 10.1016/j.envres.2005.08.009 |
| Lee et al. | 2024 | Cross-sectional observational study of 779 households in a farming cohort. | whole genome | bacteria | USA | 10.1186/s40168-024-01823-y |
| Lee et al. | 2021a | Case-control study of house dust microbiota in adults with asthma, atopy, and hay fever. | 16S | bacteria | USA | 10.1016/j.jaci.2020.06.013 |
| Lee et al. | 2021b | Cross-sectional study collecting air samples in schools to assess indoor air quality in classrooms. | 16S and ITS | bacteria, fungi | South Korea | 10.1111/ina.12825 |
| Leung et al. | 2008 | Cross-sectional study of respiratory diseases with Staphylococcus aureus in dust. | femB gene | bacteria | USA | 10.1111/j.1365-2222.2008.02964.x |
| Leung et al. | 2014 | Cross-sectional study of diversity and dynamics of indoor-air microbiomes in a subway network. | 16S (V4) | bacteria | China (Hong Kong) | 10.1128/AEM.02244-14 |
| Leung et al. | 2017 | Longitudinal observational study of airborne bacterial communities in a zero-carbon building. | 16S (V4) | bacteria | China (Hong Kong) | 10.1111/ina.12410 |
| Li et al. | 2013 | Cross-sectional study analyzing AC and engine filter dust samples from vehicles for biological content like bacteria and fungi. | Not performed | bacteria, fungi | China | 10.1021/es402848d |
| Li et al. | 2022 | Cross-sectional study of microbial community structures and potential bacterial pathogens in outdoor dust of kindergartens. | 16S (V3–V4) | bacteria, fungi | China | 10.1016/j.envint.2022.107577 |
| Loo et al. | 2018 | Cross-sectional study of microbiota and allergen profiles in house dust from allergic and non-allergic subjects. | 16S (V3-4) | bacteria | Singapore | 10.1186/s40413-018-0212-5 |
| Ludwig et al. | 2017 | Cross-sectional study of Staphylococcus aureus and staphylococcal enterotoxins in homes of inner-city adults with asthma. | Not performed | bacteria | USA | 10.1016/j.scitotenv.2017.01.003 |
| Maestre et al. | 2018 | Cross-sectional study evaluating the effect of different dust collection methods on microbial diversity and richness. | 16S and ITS | bacteria, fungi | USA | 10.1186/s40168-018-0407-6 |
| Mäki et al. | 2021 | Cross-sectional study between dog keeping and indoor dust microbiota. | 16S (V3–V4) and ITS1 | bacteria, fungi | Finland and Germany | 10.1038/so'co41598-021-84790-w |
| Martikainen et al. | 2021 | Cross-sectional and Experimental laboratory study Microbiological characterization of cow stable dust. | 16S and ITS | bacteria, fungi | Finland | 10.1016/j.tiv.2021.105202 |
| Martin-Sanchez et al. | 2021 | Cross-sectional study of indoor mycobiomes in 271 houses. | ITS | fungi | Norway | 10.1111/mec.15916 |
| Nastasi et al. | 2020 | Cross-sectional and Experimental laboratory study comparing the fungal growth quantity and morphology in residential carpet under different environmental conditions. | 18S | fungi | USA | 10.1016/j.buildenv.2020.106774 |
| Niemeier-Walsh et al. | 2021 | Prospective cohort study comparing mycobiomes and bacteriomes between home dust, saliva, and sputum samples. | 16S (V3–V4) and ITS1 | bacteria, fungi | USA | 10.1111/ina.12750 |
| Noris et al. | 2011 | Longitudinal observational study evaluating HVAC filters as a sampling mechanism for indoor microbial communities. | 16S and ITS | bacteria, fungi | USA | 10.1016/j.atmosenv.2010.10.017 |
| Núñez & García | 2022 | Cross-sectional study of the effect of natural passive ventilation on bioaerosol concentrations, comparing indoor and outdoor samples. | 16S and ITS | bacteria, fungi | Spain | 10.1016/j.buildenv.2021.108438 |
| Nygaard & Charnock | 2018 | Longitudinal observational study assessing changes in dust microbiomes over time in newly opened kindergartens. | 16S | bacteria | Norway | /10.1186/s40168-018-0553-x |
| O’Connor, et al. | 2018 | Prospective cohort study observing early-life home environment and the risk of developing asthma among inner-city children. | 16S | bacteria | USA | 10.1016/j.jaci.2017.06.040 |
| Onwusereaka et al. | 2024 | Cross-sectional comparative study of indoor and outdoor dust samples from preschools exposed to air pollutants. | ITS1 | bacteria, fungi | Malaysia | 10.1007/s11869-024-01545-y |
| Park et al | 2021 | Cross-sectional study collecting floor dust from elementary schools for microbial composition analysis. | 16S (V5-7) | bacteria | USA | 10.1186/s40168-020-00954-2 |
| Park et al. | 2022 | Cross-sectional study characterizing fungal communities in classroom floor dust. | ITS | fungi | USA | 10.1021/acs.est.2c01703 |
| Richardson et al. | 2019 | Cross-sectional study observing microbiome and allergens in bedrooms of patients suffering from allergy. | 16S | bacteria | USA | 10.1186/s40168-019-0695-5 |
| Rittenour et al. | 2014 | Cross-sectional study of fungal diversity in homes of asthmatic children. | ITS | fungi | USA | 10.1039/c3em00441d |
| Rocchi et al. | 2015 | Prospective cohort study measuring microorganism composition from electrostatic dust collectors in French child cohort homes. | Not performed | bacteria, fungi | France | 10.1016/j.scitotenv.2014.10.086 |
| Ross et al. | 2000 | Longitudinal observational study (panel study) of house dust-mite allergens, bacteria, mold spores, and asthma symptoms in flood-affected areas. | Not performed | bacteria, fungi | USA | 10.1034/j.1398-9995.2000.00551.x |
| Sautour et al. | 2009 | Prospective comparative study collecting indoor and outdoor air samples at a hospital using a mobile air-decontamination unit. | ITS1 | fungi | France | 10.1016/j.scitotenv.2009.02.024 |
| Shabankarehfard et al. | 2017 | Matched case-control study evaluating associations of allergic diseases with fungi concentrations in air and dust. | Not performed | fungi | Iran | 10.1080/08820139.2017.1322102 |
| Shan et al. | 2020 | Cross-sectional study investigating bacterial diversity in house dust from regions differing in urbanization and pet ownership. | 16S (V3–V4) | bacteria | China and Australia | 10.1016/j.waojou.2020.100452 |
| Sitarik et al. | 2018 | Longitudinal observational study assessing dust microbiome changes with the introduction of a dog into the home. | 16S | bacteria | USA | 10.1111/ina.12456. |
| Sun et al. | 2022 | Repeated cross-sectional study of asthma, rhinitis, and eczema in preschool children and their interactions with classroom dust microbiomes. | 16S and ITS | bacteria, fungi | China | 10.1016/j.envint.2022.107137 |
| Šarac et al. | 2025 | Cross-sectional study investigating the relationship between environmental and household factors and indoor bacterial communities in households. | 16S | bacteria | Croatia | 10.1080/03014460.2025.2509606 |
| Šunić et al. | 2025 | Cross-sectional study investigating the relationship between environmental and household factors and indoor fungal communities in households. | ITS2 | fungi | Croatia | 10.3390/jof11040261 |
| Ta¨ubel et al. | 2009 | Cross-sectional study comparing bacterial diversity and composition in house dust and skin swabs to investigate human bacterial contributions. | 16S | bacteria | Finland | 10.1016/j.jaci.2009.07.045 |
| Tang et al. | 2024 | Case-control study of 62 allergic rhinitis and 51 healthy children. | 16S | bacteria | China | 10.1186/s12866-024-03668-9 |
| Tischer et al. | 2016 | Prospective cohort study measuring exposure to fungal and bacterial diversity in house dust and its effects on allergic sensitization and wheezing in childhood. | Not performed | bacteria, fungi | Germany | 10.1289/EHP158 |
| Tong et al. | 2017 | Cross-sectional study collecting air samples, surface swabs, and skin swabs from homes in Hong Kong. | ITS1 | fungi | Hong Kong | 10.1186/s40168-017-0346-7 |
| Valkonen et al. | 2015 | Nested case-control study collecting and analysis of mattress dust samples from children in different environmental and health categories to study microbial associations. | Not performed | bacteria | Germany, Austria, Switzerland | 10.1371%2Fjournal.pone.0131594 |
| Valkonen et al. | 2018 | Case-control study analysing mattress dust samples from asthmatics and control subjects in European countries to study microbial levels and asthma associations. | Not performed | bacteria, fungi | Germany, Spain, France, UK, Iceland, Sweden, Switzerland | 10.1111/ina.12427 |
| Vandenborght et al. | 2021 | Prospective cohort study analysing microbiome and mycobiome analysis of patients with severe asthma, including dust and sputum samples. | 16S and ITS | bacteria, fungi | France | 10.1016%2Fj.jaci.2020.08.035 |
| Veillette et al. | 2013 | Cross-sectional study evaluating bioaerosol emissions from various vacuum cleaners. | Not performed | bacteria, fungi | Australia | 10.1128/AEM.01583-13. |
| Vestergaard et al. | 2018 | Cross-sectional study analysing indoor dust samples from farmers’ homes, pig stables, and suburban homes for bacterial community studies. | 16S (V3–V4) | bacteria | Denmark | 10.3389/fmicb.2018.00870 |
| Vidal-Quist et al. | 2021 | Experimental laboratory study characterizing RNA viruses from Dermatophagoides pteronyssinus and their detection in mite-derived sources. | Not performed | viruses | Spain | 10.1111/all.14884 |
| Wang et al. | 2023 | Nested case-control study of the influence of farm exposures on house dust microbiome. | Not performed | bacteria | USA | 10.3389/fmicb.2023.1202194 |
| Weikl et al. | 2016 | Cross-sectional study of the influence of environmental factors on fungal and bacterial communities. | 16S and ITS | bacteria, fungi | Germany | 10.1371/journal.pone.0154131 |
| Yamamoto et al. | 2011 | Longitudinal observational study assessing allergic fungi content in floor dust using floor wipe sampling and qPCR. | Not performed | fungi | USA | 10.1111/j.1600-0668.2011.00732.x |
| Yang et al. | 2022 | Cross-sectional study of the effect of disinfection and environmental factors on airborne microbial communities in schools during COVID. | 16S and ITS | bacteria, fungi | Korea | 10.1111/ina.13107 |
| Zhao et al. | 2025 | Prospective repeated-measure study of indoor airborne microbiome in university dormitories. | 16S and ITS2 | bacteria, fungi | China | 10.1016/j.jes.2025.04.022 |
| Zhou et al. | 2021 | Cross-sectional study of polluted outdoor air on indoor airborne microbiomes. | 16S and ITS | bacteria, fungi | China | 10.1016/j.jaerosci.2021.105798 |


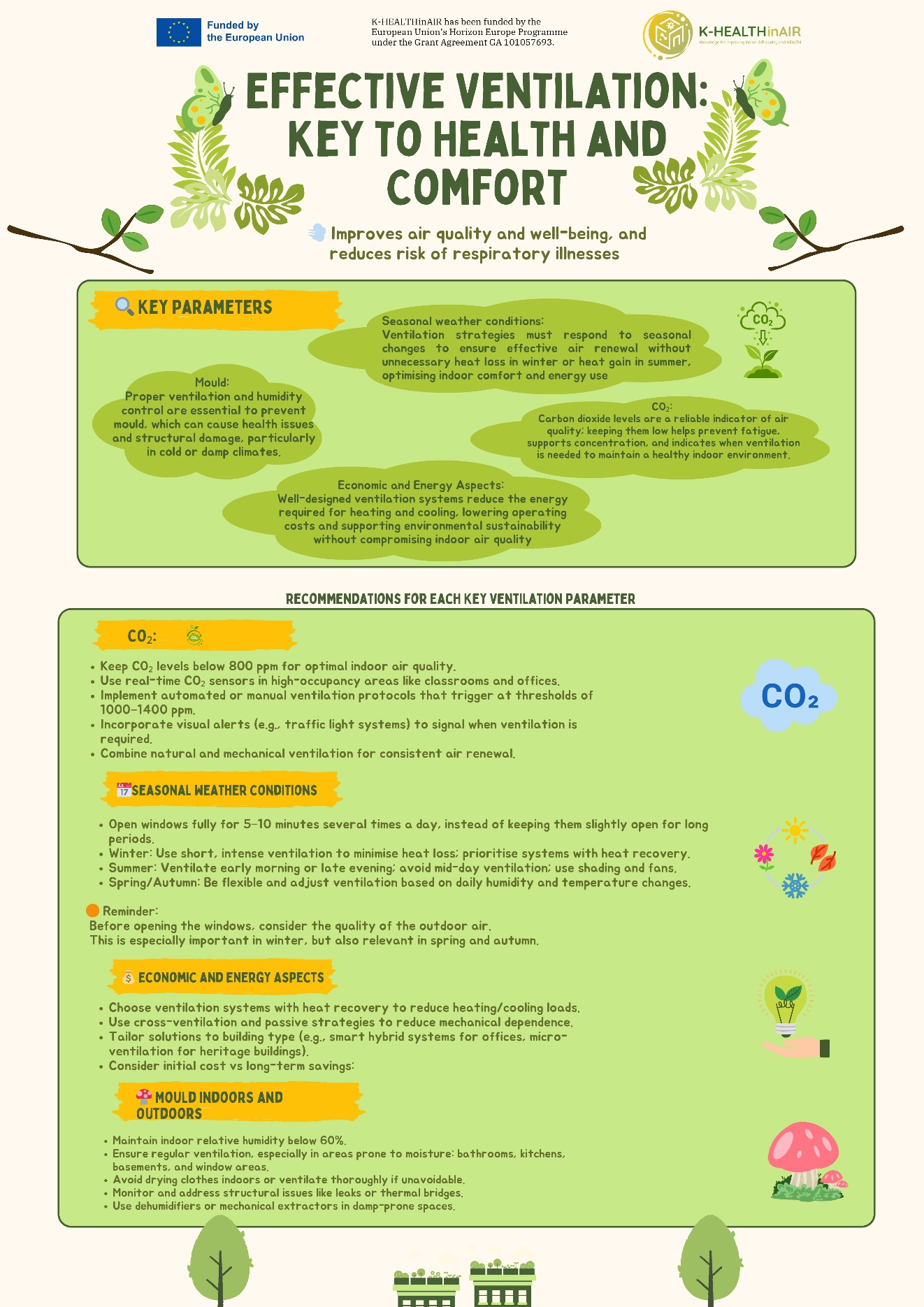
Figure S1. Effective ventilation: key to health and comfort. Infographic prepared as part of the K-HEALTHinAIR project to raise public awareness on reducing the risk of respiratory illnesses by improving indoor air quality (IAQ).
